# Resistome, virulome and genomic insights into the silent dissemination of *Acinetobacter baumannii* during the COVID-19 pandemic in Brazil

**DOI:** 10.3389/fcimb.2026.1857994

**Published:** 2026-06-29

**Authors:** Felipe Ramos Pinheiro, Renata Freire Alves Pereira, Giorgio Silva-Santana, André Luis Canellas, Bruno de Araújo Penna, Thiago Pavoni Gomes Chagas, Fabio Aguiar-Alves

**Affiliations:** 1 Laboratory of Molecular Epidemiology and Biotechnology, School of Pharmacy, Fluminense Federal University, Niteroi, Brazil; 2 Graduate Program in Pathology, Fluminense Federal University, Niteroi, Brazil; 3 Graduate Program in Microbiology and Parasitology, Biomedical Institute - Fluminense Federal University, Niteroi, Brazil; 4 Department of Experimental and Preclinical Development, Bio-Manguinhos, Oswaldo Cruz Foundation (Fiocruz), Rio de Janeiro, Brazil; 5 Laboratory of Gram-Positive Cocci, Fluminense Federal University, Niteroi, Brazil; 6 Department of Pathology, School of Medicine, Fluminense Federal University, Niteroi, Brazil; 7 Department of Pharmaceutical Sciences, Lloyd L. Gregory School of Pharmacy, Palm Beach Atlantic University, West Palm Beach, FL, United States

**Keywords:** *Acinetobacter baumannii*, COVID-19, genomic surveillance, resistome, virulome

## Abstract

**Introduction:**

*Acinetobacter baumannii* is a Gram-negative opportunistic pathogen frequently associated with severe nosocomial infections, particularly in immunocompromised patients. Its remarkable ability to acquire and disseminate antimicrobial resistance mechanisms promotes its persistence in hospital environments, representing a major challenge for infection control.

**Methods:**

This study aimed to characterize clinical *A. baumannii* strains recovered from colonization samples of patients with laboratory-confirmed SARS-CoV-2 infection in Brazil between May 2021 and April 2022, focusing on antimicrobial resistance profiles, virulence determinants, and genomic diversity. A total of 16 clinical strains obtained from nasopharyngeal, tracheal, and blood samples were analyzed. The strains were identified by MALDI-TOF mass spectrometry, subjected to antimicrobial susceptibility testing by disk diffusion, and subsequently characterized by whole-genome sequencing.

**Results:**

Among the 16 strains analyzed, 15 (93.8%) were classified as multidrug-resistant (MDR) and exhibited carbapenem resistance, being classified as carbapenem-resistant A. baumannii (CRAB). Resistance to imipenem and meropenem was observed in 93.8% (n=15) and 87.5% (n=14) of the strains, respectively. Whole-genome sequencing revealed the presence of important resistance determinants, including *blaOXA-23-like*, *armA*, *tet(B)*, and *mph(E)/msr(E)*. Analysis of virulence factors identified genes associated with pilus formation and cellular adhesion, a complete pga operon involved in biofilm production in most strains, as well as widely distributed quorum sensing systems and iron acquisition mechanisms.

**Discussion:**

Collectively, the results demonstrate the widespread dissemination of MDR/CRAB *A. baumannii* lineages during the COVID-19 pandemic in Brazil, supported by a diverse repertoire of genetic determinants associated with antimicrobial resistance and virulence. These findings highlight the importance of continuous genomic surveillance and strengthened infection prevention and control strategies to mitigate the risk of hospital outbreaks caused by this pathogen.

## 1 Introduction

*Acinetobacter baumannii* is recognized as an opportunistic pathogen associated with severe infections in hospital settings, particularly in immunocompromised patients or those in Intensive Care Units (ICUs) (Ahuatzin-Flores et al., 2024; França et al., 2025). The World Health Organization (WHO) includes *A. baumannii*, particularly carbapenem-resistant strains (CRAB), in the Priority Pathogens List of Critical Concern due to its extensive antimicrobial resistance, high clinical impact, and limited therapeutic options (WHO, 2024).

Clinical and epidemiological studies have shown that *A. baumannii* is strongly associated with nosocomial infections, being prevalent in ventilator-associated pneumonia (VAP) in ICUs. Additionally, this pathogen causes bacteremia, catheter-associated urinary tract infections, and wound infections, particularly in critically ill patients (Lima et al., 2019; Zhang et al., 2021; Ahuatzin-Flores et al., 2024).

Beyond clinical settings, *Acinetobacter* species have been isolated from natural environments, including water and environmental surfaces, indicating a broad ecological distribution. Their persistence outside hospitals suggests that environmental reservoirs may contribute to cross-transmission, particularly when hygiene or water treatment measures are inadequate (Carvalheira et al., 2021; Gheorghe-Barbu et al., 2024).

A key factor contributing to the persistence of *A. baumannii* is its ability to form biofilms (Shenkutie et al., 2020; Silva-Santana et al., 2025a). Biofilm formation is associated with survival under adverse environmental conditions; increased tolerance to antimicrobials; persistence on hospital surfaces, facilitating nosocomial dissemination; and difficulty of eradication by host immune responses and antibiotic treatments (Mohamed et al., 2023; Choudhary and Shariff, 2025; Silva-Santana et al., 2025b). *A. baumannii* biofilms are linked to persistent tissue colonization and adherence to invasive medical devices, representing one of the main obstacles to effective infection control (Roy et al., 2022).

In this study, we characterized 16 clinical *A. baumannii* strains isolated from patients with laboratory-confirmed SARS-CoV-2 infection at Antônio Pedro University Hospital between May 2021 and April 2022. The isolates were recovered from colonization samples and were not associated with healthcare-associated infections. We investigated their antimicrobial resistance profiles, virulence determinants, and genomic diversity using whole-genome sequencing (WGS).

## 2 Materials and methods

### 2.1 Bacterial strains

A total of 598 bacterial strains were analyzed, isolated from patients attended at the Multiuser Laboratory for Research Support in Nephrology (LAMAP) and the Clinical Microbiology Laboratory of Antônio Pedro University Hospital (HUAP), both affiliated with Fluminense Federal University (UFF), in Niterói, Rio de Janeiro, Brazil, between May 2021 and April 2022 during the COVID-19 pandemic. The isolates originated from patients admitted to critical hospital sectors, including the Emergency Department and ICU, which are healthcare environments characterized by high patient complexity and increased antimicrobial selective pressure.

Among these isolates, 68 strains were identified as *A. baumannii* and recovered from colonization samples obtained from patients with confirmed SARS-CoV-2 infection. The isolates did not correspond to healthcare-associated infections (HAIs). Each strain analyzed in this study originated from a different patient, ensuring epidemiological independence among isolates and minimizing the possibility of duplicate sampling.

Of these 68 A*. baumannii* strains, 15 multidrug-resistant (MDR) isolates were selected for in-depth characterization, and one fully susceptible isolate was included as a comparative control for detailed whole-genome sequencing analyses in the present study.

All strains were stored at −80 °C in Brain Heart Infusion (BHI) medium (Difco Laboratories^®^, Detroit, MI, USA) supplemented with 10% (v/v) glycerol at the Laboratory of Molecular Epidemiology and Biotechnology (LEMB) of UFF.

The study was approved by the Research Ethics Committee of the School of Medicine of Fluminense Federal University under the Certificate of Presentation for Ethical Appraisal (CAAE) No. 26823614.2.0000.5243.

### 2.2 Species identification by MALDI-TOF MS

Species identification was performed by matrix-assisted laser desorption/ionization time-of-flight mass spectrometry (MALDI-TOF MS) using the Microflex LT^®^ system (Bruker Daltonics, Bremen, Germany). Spectral analysis was conducted using the MALDI Biotyper^®^ software (version 1.2), following the manufacturer’s instructions. Score interpretation followed the criteria recommended by Bruker Daltonics^®^: values between 0.00 and 1.69 were considered indicative of unreliable identification; values between 1.70 and 1.99 indicated reliable identification at the genus level, but with low confidence for species-level identification; and values between 2.00 and 3.00 were considered highly reliable for identification at both genus and species levels.

### 2.3 Antimicrobial resistance profile

Antimicrobial resistance was assessed by antimicrobial susceptibility testing (AST) using the disk diffusion method on Mueller–Hinton agar (MHA) (Difco Laboratories^®^), in accordance with the Clinical and Laboratory Standards Institute (CLSI) guidelines, M100, 33rd edition (CLSI, 2023). The tested antimicrobials (Oxoid™, UK) included amikacin (AMK, 30 µg), ampicillin/sulbactam (AMP/SUL, 10/10 µg), piperacillin/tazobactam (TZP, 100/10 µg), ceftazidime (CAZ, 30 µg), imipenem (IPM, 10 µg), and meropenem (MEM, 10 µg). The MDR profile was defined as acquired resistance to at least one antimicrobial agent in three or more distinct antimicrobial classes, according to the criteria proposed by Magiorakos et al. (2012). In addition, isolates resistant to carbapenems (IPM and/or MEM) were classified as carbapenem-resistant *A. baumannii* (CRAB). Because several antimicrobial categories, including last-resort agents such as colistin and tigecycline, were not evaluated, formal classification as extensively drug-resistant (XDR) or pandrug-resistant (PDR) was not performed.

### 2.4 Whole-genome sequencing

Genomic DNA from the strains was extracted using the QIAamp DNA Mini^®^ kit (Qiagen, Hilden, Germany), following the manufacturer’s standard protocol, including an enzymatic lysis step with proteinase K. DNA quantification was performed by fluorometry using the QuantiFluor ONE dsDNA^®^ system (Promega, Madison, WI, USA) on a Quantus^®^, Inc. fluorometer (Promega, Madison, WI, USA), according to the manufacturer’s instructions. Genomic libraries were prepared from purified DNA using the Nextera DNA Flex^®^ kit (Illumina, San Diego, CA, USA), following the manufacturer’s recommended protocol. After normalization and dilution, sequencing was performed on the Illumina NextSeq 2000^®^ platform using paired-end reads (2 × 100 bp) with the NextSeq 1000/2000 P2^®^ kit (200 cycles) (Illumina, San Diego, CA, USA).

Raw sequencing reads were assembled *de novo* using SPAdes^®^ software (Center for Algorithmic Biotechnology, Saint Petersburg, Russia), version 4.2.0, with default parameters. Genome annotation was performed using the NCBI Prokaryotic Genome Annotation Pipeline (PGAP) following genome submission to GenBank. Antimicrobial resistance and virulence-associated genes were considered present only when they met the sequence identity and coverage criteria established by the annotation and reference databases used in the analysis. Partial matches and truncated open reading frames (ORFs) were excluded from the final dataset to avoid overestimation of resistome and virulome profiles. Hypothetical proteins predicted during the automated annotation process were not included in downstream comparative analyses unless a putative function associated with antimicrobial resistance or virulence could be reliably assigned based on curated database-supported annotation. The genomic sequences were deposited in GenBank under the accession numbers listed in **Table 1**.

**Table 1:** Characterization and identification of *A. baumannii* isolates by MALDI-TOF MS and whole-genome sequencing (WGS).

Samples	ID	Isolation site	MALDI-TOF MS		WGS			
			Score	Consistency	GenBank ID	Completeness (%)	Contamination (%)	Genome size (bp)
332	010	TA	2.15	A	JBUCUO000000000	100.00	0.00	4, 058, 226
333	012	TA	2.31	A	JBUCUP000000000	97.39	0.64	4, 153, 145
334	027	NPS	2.24	B	JBUCUQ000000000	100.00	0.00	3, 920, 857
336	074	NPS	2.42	A	JBUCUR000000000	99.98	0.63	3, 884, 196
337	075	NPS	2.45	A	JBUCUS000000000	99.98	0.63	3, 913, 045
338	076	NPS	2.39	A	JBUCUT000000000	100.00	0.00	4, 063, 417
339	130	BC	2.22	B	JBUCUU000000000	100.00	0.00	4, 256, 491
340	131	NPS	2.11	A	JBUCUV000000000	100.00	0.05	3, 826, 120
341	132	NPS	2.33	A	JBUCUW000000000	100.00	0.59	4, 208, 756
342	182	TA	2.20	A	JBUCUX000000000	99.98	0.63	3, 918, 986
343	266	NPS	2.27	B	JBUCUY000000000	100.00	0.27	3, 906, 516
345	306	TA	2.14	A	JBUCUZ000000000	99.59	0.63	3, 822, 253
349	504	NPS	2.23	A	JBUCVA000000000	99.98	0.63	3, 941, 051
350	509	NPS	2.12	A	JBUCVB000000000	100.00	0.27	3, 892, 660
351	514	NPS	2.16	A	JBUCVC000000000	100.00	0.00	4, 129, 408
352	720	TA	2.28	A	JBUCVD000000000	99.98	0.63	3, 928, 938

Matrix-assisted laser desorption/ionization–time of flight mass spectrometry (MALDI-TOF MS) identification scores are interpreted according to the manufacturer’s criteria, where values ≥ 2.00 indicate highly reliable identification at the genus and species level, and consistency refers to the confidence category of the identification result (A = highly reliable identification; B = secure genus-level identification). Whole-genome sequencing (WGS) data include genome assemblies generated from Illumina short-read sequencing. Genome completeness (%) and contamination (%) represent quality metrics obtained from genome assembly assessment tools, indicating the proportion of recovered genomic content and potential contamination, respectively. Genome size (bp) corresponds to the total length of assembled contigs for each isolate. All isolates were identified as *A. baumannii*.

ID, identification code; BC, blood culture; NPS, nasopharyngeal swab; TA, tracheal aspirate; WGS, whole-genome sequencing.

The assembled genomes were analyzed using the Proksee v1.0.2 platform (University of Alberta, Edmonton, Alberta, Canada) for visualization and comparative annotation. The reference strains employed were *A. baumannii* ATCC 17978 and AB5075-UW (GenBank accession numbers CP000521.1 and CP008706.1). Genomic similarity was assessed through BLAST alignments using the BLAST+ package (v2.17.0), enabling the identification of conserved and divergent regions relative to the reference genomes. The results were visualized in a linear genomic map displaying the alignment regions distributed across the chromosome (Grant et al., 2023).

The identification of antimicrobial resistance-associated genes was performed using the Resistance Gene Identifier (RGI; v6.0.5), based on the Comprehensive Antibiotic Resistance Database (CARD; v4.0.1). The detected genes were mapped throughout the genome to evaluate the distribution of resistance determinants. Additionally, GC content and GC skew plots were generated using the integrated tools available in Proksee to identify potential regions acquired through horizontal gene transfer and to assess the structural integrity of the genome assembly (Alcock et al., 2023; Grant et al., 2023).

### 2.5 Statistical analysis

The presence of antimicrobial resistance genes and virulence factors was coded as a binary variable (0 = absent; 1 = present). For each gene, the mean occurrence among the strains was calculated and subsequently expressed as a percentage, and the standard deviation (SD) was used to estimate variability among the strains. Analyses were conducted separately for antimicrobial resistance determinants, stratified by class: aminoglycosides (AGs), β-lactams, macrolide–lincosamide–streptogramin (MLS), tetracyclines (TCs), chloramphenicol (CHL), and sulfonamides/trimethoprim (SXT). Similarly, virulence factors were grouped into the following functional categories: pili/adhesins (PA), biofilm formation (BF), quorum sensing (QS), toxins/enzymes (TE), iron acquisition/siderophores (SID), and efflux systems (ESs). Statistical analyses were performed using GraphPad Prism^®^ software, version 9.5.1.

## 3 Results

### 3.1 Genomic characterization, identification, and quality assessment

All 16 strains were confirmed as *A. baumannii* by MALDI-TOF MS. The isolates were recovered from nasopharyngeal samples (62.5%; n = 10), tracheal samples (31.3%; n = 5), and blood samples (6.3%; n = 1) (**Table 1**). The predominance of isolates recovered from nasopharyngeal specimens reflects the characteristics of the study population and the surveillance strategy adopted during the COVID-19 pandemic, being consistent with the frequent detection of *A. baumannii* colonization among hospitalized patients.

WGS of the 16 strains resulted in an average coverage of approximately 260× per genome, with depth ranging from ~100× to ~360×. This depth was considered adequate for genome assembly, functional annotation, and subsequent comparative analyses (**Table 1**). The lengths of the assembled draft genomes ranged from 3, 822, 253 bp (strain 306) to 4, 256, 491 bp (strain 130), consistent with typical variability observed in draft genome assemblies generated from short-read sequencing data. Most genomes were approximately 3.8–4.2 Mbp in size, consistent with the range reported for *A. baumannii* in complete genomes deposited in public databases (NCBI RefSeq/GenBank). The 130 and 132 strains stood out for having relatively larger genomes (> 4.2 Mbp), suggesting the possible presence of additional accessory genetic content (**Table 1**).

The comparative analysis of the clinical strain genomes using the reference *A. baumannii* ATCC 17978 (CP000521.1) and AB5075-UW (CP008706.1) strains revealed a high degree of core genome conservation in both comparisons. BLAST alignments demonstrated extensive coverage across most of the chromosome, indicating that the isolates share the characteristic genomic architecture of the species. However, important differences were observed in the conservation patterns relative to each reference strain.

When compared to ATCC 17978, the analyzed draft genomes exhibited a greater number of regions with reduced alignment coverage and nucleotide similarity. Although these regions likely correspond, at least in part, to accessory genomic content such as genomic islands, insertion sequences, transposons, prophages, and other mobile genetic elements, we cannot exclude the contribution of assembly fragmentation inherent to short-read draft genomes.

In contrast, the comparison with the AB5075-UW strain revealed more continuous alignments and greater structural conservation throughout the chromosome. A smaller number of divergent regions was observed, along with a higher correspondence between genomic segments containing antimicrobial resistance-associated genes. The resistance determinants identified by the RGI/CARD system displayed a distribution pattern similar to that observed in AB5075-UW, frequently located within chromosomal regions conserved among the compared genomes.

The GC content and GC skew profiles displayed patterns consistent with complete and structurally intact bacterial chromosomes in both analyses. However, the regions that exhibited divergence relative to ATCC 17978 showed greater correspondence with conserved segments in AB5075-UW, reinforcing the hypothesis that these regions represent accessory genome components frequently found in contemporary clinical lineages of *A. baumannii*.

**Figure 1:**
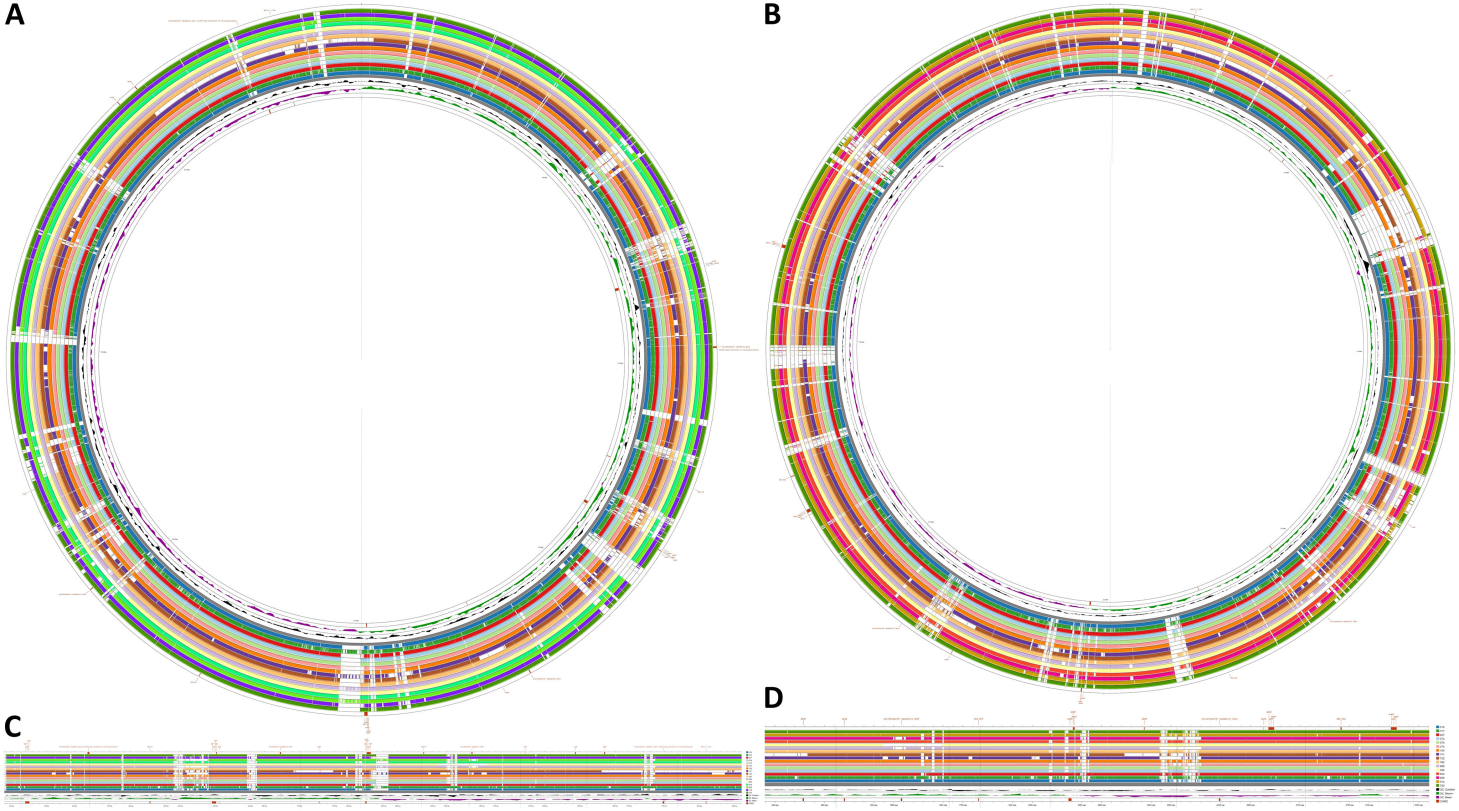
Comparative genomic alignment and structural characterization of *A. baumannii* genomes using the reference strains AB5075-UW and ATCC 17978. Comparative genomic analyses were performed using the Proksee v1.0.2 platform for genome visualization and comparative annotation. Panels A and C represent the alignment of assembled *A. baumannii* genomes against the reference strain AB5075-UW (GenBank accession no. CP008706.1), whereas panels B and D represent the alignment against the reference strain ATCC 17978 (GenBank accession no. CP000521.1). Circular genomic maps **(A, B)** and linear alignment representations **(C, D)** were generated based on sequence comparisons using BLAST+ (v2.17.0), highlighting conserved and divergent genomic regions among the analyzed strains. Antimicrobial resistance-associated genes identified using the Resistance Gene Identifier (RGI v6.0.5) based on the Comprehensive Antibiotic Resistance Database (CARD v4.0.1) were mapped throughout the genomes. The inner rings correspond to GC content and GC skew analyses, which were used to identify potential horizontally acquired regions and evaluate the structural integrity of the genome assemblies. White regions indicate areas of low similarity or absence of alignment relative to the reference genomes. (National Center for Biotechnology Information (NCBI), 2025a, 2025b).

The results indicate that the analyzed clinical strains exhibit greater genomic similarity to the AB5075-UW strain than to the ATCC 17978 strain. This pattern suggests the sharing of genetic features associated with modern hospital lineages, including accessory regions related to antimicrobial resistance, adaptation to the hospital environment, and acquisition of mobile genetic elements. Considering that AB5075-UW is recognized as a hypervirulent and MDR strain widely used as a model for pathogenicity studies, these findings reinforce the hypothesis that the evaluated isolates belong to an evolutionary context more closely related to contemporary clinical lineages of *A. baumannii* than to the ATCC 17978 reference strain, which is often considered representative of a more conserved genome within the species (**Figure 1**).

### 3.2 Antimicrobial resistance phenotype

Among the 16 clinical *A. baumannii* strains analyzed, 93.8% (n = 15) were classified as MDR, showing acquired resistance to at least one antimicrobial agent in three or more distinct antimicrobial classes. A high prevalence of carbapenem resistance was observed, with 93.8% (n = 15) of the strains resistant to IPM and 87.5% (n = 14) resistant to MEM. Accordingly, 93.8% (n = 15) of the isolates were classified as CRAB. Resistance to AMK was detected in 56.3% (n = 9) of the strains. Among the β-lactam/β-lactamase inhibitor combinations, only 6.3% (n = 1) of the strains were resistant to AMP/SUL, whereas 93.8% (n = 15) were resistant to TZP. Resistance to CAZ was also detected in 93.8% (n = 15) of the strains. Notably, isolate 131 was susceptible to all antimicrobial agents tested and was the only isolate not classified as either MDR or CRAB, which explains why the major resistance phenotypes were observed in 15 of the 16 isolates analyzed (**Table 2**).

**Table 2:** Antimicrobial resistance profile of *A. baumannii* isolates.

Samples	ID	Antimicrobial test								
		AMK	AMP/SUL	TZP	CAZ	IPM	MEM	Resistant classes (n)	MDR	CRAB
332	010	R	S	R	R	R	R	4	Y	Y
333	012	S	S	R	R	R	R	3	Y	Y
334	027	I	R	R	R	R	R	4	Y	Y
336	074	R	S	R	R	R	R	4	Y	Y
337	075	S	I	R	R	R	R	3	Y	Y
338	076	R	S	R	R	R	R	4	Y	Y
339	130	R	S	R	R	R	R	4	Y	Y
340	131	S	S	S	S	S	S	0	N	N
341	132	S	S	R	R	R	R	3	Y	Y
342	182	R	S	R	R	R	R	4	Y	Y
343	266	S	S	R	R	R	R	3	Y	Y
345	306	R	S	R	R	R	R	4	Y	Y
349	504	R	I	R	R	R	R	4	Y	Y
350	509	I	S	R	R	R	R	3	Y	Y
351	514	R	S	R	R	R	I	4	Y	Y
352	720	R	S	R	R	R	R	4	Y	Y

Antimicrobial susceptibility testing was performed against representative agents from different pharmacological classes, including aminoglycosides (amikacin, AMK), β-lactam/β-lactamase inhibitor combinations (ampicillin/sulbactam, AMP/SUL; piperacillin/tazobactam, TZP), third-generation cephalosporins (ceftazidime, CAZ), and carbapenems (imipenem, IPM; meropenem, MEM). Susceptibility categories were interpreted according to standard clinical breakpoints, where R indicates resistant, S susceptible, and I intermediate. Intermediate results were considered non-susceptible for the purpose of resistance classification. Antimicrobials belonging to the same pharmacological class were considered a single class when calculating the number of resistant classes per isolate.

Multidrug-resistant (MDR) was defined as acquired resistance to at least one antimicrobial agent in three or more antimicrobial classes, according to the criteria proposed by Magiorakos et al. (2012). Carbapenem-resistant *A. baumannii* (CRAB) was defined as resistance to imipenem and/or meropenem.

ID, identification code; R, resistant; S, susceptible; I, intermediate; MDR, multidrug-resistant; CRAB, carbapenem-resistant *A. baumannii*.

### 3.3 Antimicrobial resistance genotype

**Figure 2:**
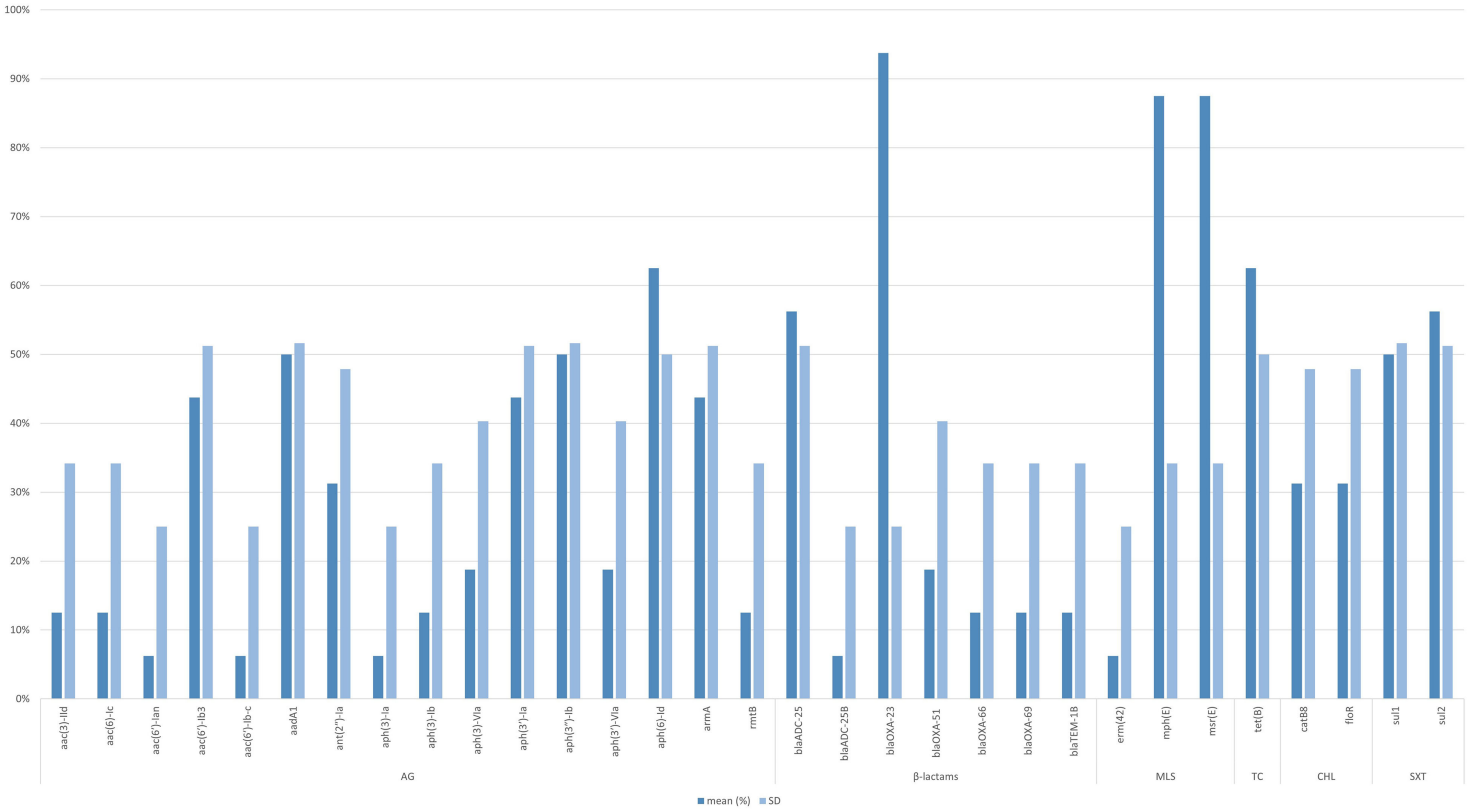
Distribution of antimicrobial resistance genes according to antimicrobial classes in clinical *A. baumannii* isolates. Bar plots represent the mean relative frequency (%) of detection of antimicrobial resistance genes identified by in silico whole-genome analysis among the analyzed *A. baumannii* isolates. Dark blue bars indicate the mean detection frequency of each resistance determinant, whereas light blue bars represent the corresponding standard deviation (SD), reflecting interstrain variability. Resistance genes are grouped according to their associated antimicrobial classes: aminoglycosides (AGs), β-lactams, macrolide–lincosamide–streptogramin (MLS), tetracyclines (TCs), chloramphenicol (CHL), and sulfonamides/trimethoprim (SXT). The figure highlights the heterogeneous distribution of resistance determinants among the analyzed strains, including genes encoding aminoglycoside-modifying enzymes, β-lactamases, efflux systems, and other acquired resistance mechanisms.

Heterogeneous resistance profiles to AGs were observed, with detection of genes encoding different mechanisms, including aminoglycoside acetyltransferases (AAC), aminoglycoside adenyltransferases (AAD), aminoglycoside phosphotransferases (APH/ANT), and 16S ribosomal RNA methylases (ARM/RMT), associated with varying levels and spectra of resistance to this class of antimicrobials. Some strains carried multiple genetic determinants, while others exhibited more restricted profiles (**Figure 2**).

The 012 and 306 strains stood out for harboring up to nine distinct genes related to AGs resistance, including *arm*A (37.5%, n = 6) and *rmt*B (12.5%, n = 2), which are associated with high-level resistance to clinically restricted AGs. In contrast, 131 strain lacked detectable genes associated with AGs resistance, suggesting phenotypic susceptibility. The most frequently detected genes included *aad*A1 (50.0%, n = 8), *aph*(3′)-Ia (43.8%, n = 7), *aph*(3″)-Ib (50.0%, n = 8), and *aph*(6)-Id (62.5%, n = 10). Other genes, such as *aac*(3)-IId, *aac*(6)-Ic, *aac*(6′)-Ib-c, *ant*(2″)-Ia, *aph*(3)-VIa, and *rmt*B, were detected less frequently, contributing to the diversity of the observed genetic profiles.

The *bla*_OXA-23-like_ gene was detected in 83.3% (n = 15) of the strains. Additional genes, such as *bla*_OXA-51-like_ (18.8%, n = 3) and *bla*_OXA-66_ (12.5%, n = 2), were identified in subsets of strains. AmpC-type β-lactamases, mediated by *bla*_ADC-25_ and *bla*_ADC-25B_, were detected in 47.1% (n = 8) and 5.9% (n = 1) of the strains, respectively. The *bla*_OXA-69_ and *bla*_TEM-1B_ genes were detected in a limited number of strains, indicating further diversification of the β-lactam resistome. The 131 strain lacked genes associated with β-lactam resistance, suggesting susceptibility to this class. Genes associated with MLS resistance were detected in 76.5% (n = 13) of the analyzed strains. The *mph*(E) gene, encoding a phosphotransferase (APH) that inactivates macrolides, and *msr*(E), encoding an ES associated with resistance to macrolides and lincosamides, were detected in 010, 012, 027, 074, 076, 130, 132, 182, 266, 306, 504, 509, and 720 strains.

Regarding tetracyclines, genomic analysis identified the *tet*(B) gene, which encodes an efflux pump associated with decreased susceptibility to tetracycline-class antibiotics. This determinant was detected in 62.5% (n = 10) of the strains (012, 074, 075, 130, 132, 182, 306, 504, 514, and 720). Because tetracycline susceptibility was not evaluated phenotypically in this study, these findings indicate the genetic potential for resistance rather than a confirmed resistance phenotype.

Genes associated with CHL resistance were detected in 56.3% (n = 9) of the strains. The *flo*R gene, associated with CHL-specific ESs, was detected in 31.3% (n = 5) of the strains (010, 076, 266, 306 and 720). The *cat*B8 gene, encoding an acetyltransferase involved in CHL inactivation, was detected in 31.3% (n = 5) of the strains (012, 182, 306 and 509). The 306 strain harbored both genes simultaneously, suggesting a potentially more robust resistance profile to this antimicrobial. Seven strains (027, 074, 075, 130, 131, 132 and 514) lacked genes associated with CHL resistance, indicating susceptibility.

Genes associated with resistance to sulfonamides and trimethoprim were detected in 93.8% (n = 15) of the strains, with variable distribution of the *sul*1 (50%; n=8) and *sul*2 (56.3%; n=9) genes, which encode insensitive variants of dihydropteroate synthase (**Table 3**).

**Table 3:** Detection of antimicrobial resistance genes in *A. baumannii* isolates.

Samples	ID	Adaptive function – resistance					
		AGs	β-lactams	MLS	TCs	CHL	SXT
332	010	*ant*(2’’)-Ia, *aph*(3)-VIa	*bla*_ADC-25_, *bla*_OXA-23-like_, *bla*_OXA-51-like_	*mph*(E), *msr*(E)	ND	*flo*R	*sul*2
333	012	*aac*(3)-IId, *aac*(6)-Ic, *aac*(6′)-Ib3, *aad*A1, *aph*(3)-Ia, *aph*(3)-Ib, *aph*(6)-Id, *arm*A, *rmt*B	*bla*_OXA-23-like_, *bla*_OXA-66_	*mph*(E), *msr*(E)	*tet*(B)	*cat*B8	*sul*1
334	027	*aph*(3′)-Via	*bla*_ADC-25_, *bla*_OXA-23-like_, *bla*_OXA-51-like_	*mph*(E), *msr*(E)	ND	ND	*sul*2
336	074	*aac*(6′)-Ib3, *aad*A1, *aph*(3)-Ib, *aph*(3′)-Ia, *aph*(6)-Id, *arm*A	*bla*_ADC-25_, *bla*_OXA-23-like_, *bla*_OXA-66_	*mph*(E), *msr*(E)	*tet*(B)	ND	*sul*1
337	075	*aac*(6′)-Ib3*, aad*A1*, aph*(3′)-Ia, *aph*(3″)-Ib, *aph*(6)-Id*, arm*A	*bla*ADC-25, *bla*_OXA-23-like_	ND	*tet*(B)	ND	*sul*1
338	076	*ant*(2″)-Ia, *aph*(3)-VIa	*bla*_OXA-23-like_, *bla*_OXA-51-like_	*mph*(E), *msr*(E)	ND	*flo*R	*sul*2
339	130	*aph*(3″)-Ib, *aph*(6)-Id	*bla*_OXA-23-like_	*mph*(E), *msr*(E)	*tet*(B)	ND	*sul*2
340	131	ND	ND	ND	ND	ND	ND
341	132	*aac*(3)-IId, *aac*(6)-Ic, *aac*(6′)-Ib-c, *aad*A1, *aph*(3′)-Ia, *aph*(3″)-Ib, *aph*(6)-Id, *rmt*B	*bla*_ADC-25_B, *bla*_OXA-23-like_	*erm*(42), *mph*(E), *msr*(E)	*tet*(B)	ND	*sul*1*, sul*2
342	182	*aac*(6′)-Ib3, *aad*A1, *aph*(3′)-Ia, *aph*(3″)-Ib, *aph*(6)-Id, *arm*A	*bla*_ADC-25_, *bla*_OXA-23-like_	*mph*(E), *msr*(E)	*tet*(B)	*cat*B8	*sul*1
343	266	*ant*(2″)-Ia, *aph*(3′)-VIa	*bla*_OXA-23-like_, *bla*_OXA-69_	*mph*(E), *msr*(E)	ND	*flo*R	*sul*2
345	306	*aac*(6′)-Ib3, *aad*A1, *ant*(2″)-Ia, *aph*(3′)-Ia, *aph*(3″)-Ib, *aph*(3′)-VIa, *aph*(6)-Id, *arm*A	*bla*_ADC-25_, *bla*_OXA_-_23-like_	*mph*(E), *msr*(E)	*tet*(B)	*cat*B8, *flo*R	*sul*1, *sul*2
349	504	*aac*(6′)-Ib3, *aad*A1, *aph*(3′)-Ia, *aph*(3″)-Ib, *aph*(6)-Id, *arm*A	*bla*_ADC-25_, *bla*_OXA-23-like_	*mph*(E), *msr*(E)	*tet*(B)	*cat*B8	*sul*1
350	509	*ant*(2″)-Ia, *aph*(3)-VIa	*bla*_OXA-23-like_, *bla*_OXA-69_	*mph*(E), *msr*(E)	ND	*flo*R	*sul*2
351	514	*aac*(6′)-Ian, *aph*(3″)-Ib, *aph*(6)-Id	*bla*_OXA-23-like_, *bla*_TEM-1B_	ND	*tet*(B)	ND	*sul*2
352	720	*aac*(6′)-Ib3, *aad*A1, *aph*(3′)-Ia, *aph*(3″)-Ib, *aph*(6)-Id, *arm*A	*bla*_ADC-25_, *bla*_OXA-23-like_	*mph*(E), *msr*(E)	*tet*(B)	*cat*B8	*sul*1

Antimicrobial resistance genes were identified through in silico analysis of whole-genome sequences of *A. baumannii* isolates. Only genes meeting the sequence identity and coverage thresholds defined by the annotation pipeline were considered present. Partial hits and truncated open reading frames (ORFs) were excluded from the final dataset to avoid overestimation of resistance determinants.

Genes were categorized according to their functional association with antimicrobial resistance classes, including aminoglycosides (AGs), β-lactams (β-lactam antibiotics and carbapenems), macrolide–lincosamide–streptogramin (MLS), tetracyclines (TCs), chloramphenicol (CHL), and sulfonamides/trimethoprim (SXT). “ND” indicates that no resistance gene was detected for the corresponding antimicrobial class in the analyzed genome.

AGs, aminoglycosides; MLS, macrolide–lincosamide–streptogramin; TCs, tetracyclines; CHL, chloramphenicol; SXT, sulfonamides/trimethoprim; ND, not detected.

### 3.4 Virulence genotype

**Figure 3:**
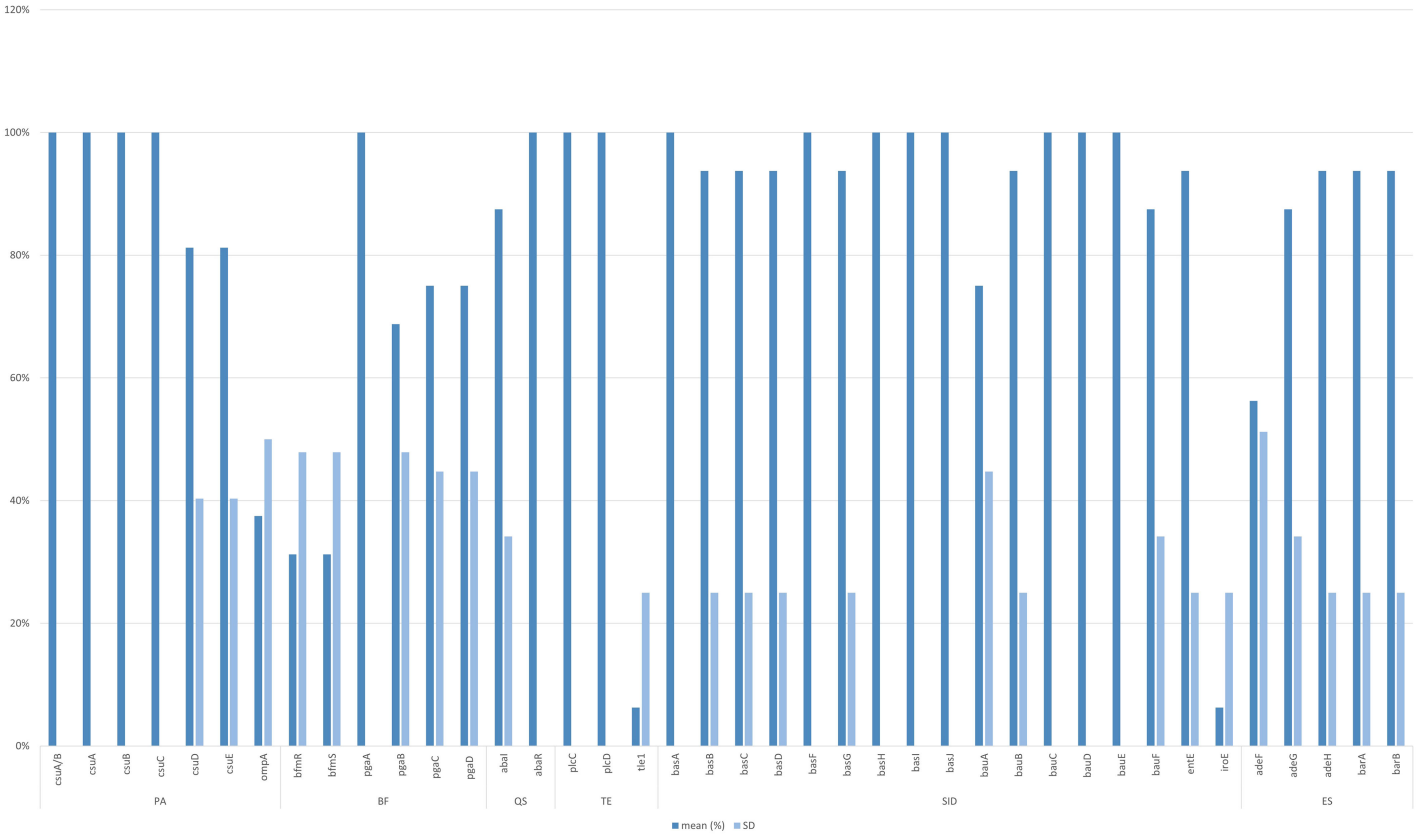
Distribution of virulence-associated genes according to functional categories in clinical *A. baumannii* isolates. Bar plots represent the mean relative frequency (%) of detection of virulence-associated genes identified by in silico whole-genome analysis among the analyzed A. baumannii isolates. Dark blue bars indicate the mean detection frequency of each virulence determinant, whereas light blue bars represent the corresponding standard deviation (SD), reflecting variability among strains. Virulence determinants were grouped according to their biological functions, including pili/adhesins (PA), biofilm formation (BF), quorum sensing (QS), toxins/enzymes (TE), iron acquisition/siderophore systems (SID), and efflux systems (ES). The figure demonstrates the broad distribution of genes associated with adhesion, biofilm establishment, bacterial communication, iron acquisition, secretion systems, and adaptive survival mechanisms, highlighting the complex virulence repertoire observed among the analyzed isolates.

In-silico analysis of the WGS data enabled the detection of genetic determinants associated with virulence in the clinical *A. baumannii* (**Table 4**; **Figure 3**).

**Table 4:** Detection of virulence genes in *A. baumannii* isolates.

Samples	ID	Biological functions – virulence					
		PA	BF	QS	TE	SID	ESs
332	010	*csu*A/B, *csu*A-C	*pga*A-C	*aba*I, *aba*R	*plc*C, *plc*D	*bas*A-D, *bas*F-J, *bau*B-E, *ent*E	*ade*F, *ade*G, *bar*A, *bar*B
333	012	*csu*A/B, *csu*A-C	*bfm*R, *bfm*S, *pga*A	*aba*I, *aba*R	*plc*C, *plc*D	*bas*A, *bas*F, *bas*H-J, *bau*A, *bau*C-E, *ent*E	*ade*F, *ade*H
334	027	*csu*A/B, *csu*A-E, *omp*A	*bfm*R, *bfm*S, *pga*A	*aba*I, *aba*R	*plc*C, *plc*D	*bas*A-D, *bas*F-J, *bau*B-F, *ent*E	*ade*F-H, *bar*A, *bar*B
336	074	*csu*A/B, *csu*A-E	*pga*A-D	*aba*I, *aba*R	*plc*C, *plc*D	*bas*A-D, *bas*F-J, *bau*A-F, *ent*E	*ade*F-H, *bar*A, *bar*B
337	075	*csu*A/B, *csu*A-E, *omp*A	*pga*A-D	*aba*I, *aba*R	*plc*C, *plc*D	*bas*A-D, *bas*F-J, *bau*A-F, *ent*E	*ade*F-H, *bar*A, *bar*B
338	076	*csu*A/B, *csu*A-D, *omp*A	*pga*A-D	*aba*I, *aba*R	*plc*C, *plc*D	*bas*A-D, *bas*F-J, *bau*A-E, *ent*E	*ade*F-H, *bar*A, *bar*B
339	130	*csu*A/B, *csu*A-E, *omp*A	*bfm*R, *bfm*S, *pga*A, *pga*D	*aba*R	*plc*C, *plc*D	*bas*A-D, *bas*F-J, *bau*B-F, *ent*E	*ade*F-H, *bar*A, *bar*B
340	131	*csu*A/B, *csu*A-E, *omp*A	*bfm*R, *bfm*S, *pga*A, *pga*C, *pga*D	*aba*R	*plc*C, *plc*D	*bas*A-D, *bas*F-J, *bau*B-F, *ent*E	*ade*F-H, *bar*A, *bar*B
341	132	*csu*A/B, *csu*A-E, *omp*A	*bfm*R, *bfm*S, *pga*A-D	*aba*I, *aba*R	*plc*C, *plc*D, *tle*1	*bas*A-D, *bas*F-J, *bau*B-F, *iro*E	*ade*F, *ade*H, *bar*A, *bar*B
342	182	*csu*A/B, *csu*A-E	*pga*A-D	*aba*I, *aba*R	*plc*C, *plc*D	*bas*A-D, *bas*F-J, *bau*A-F, *ent*E	*ade*G, *ade*H, *bar*A, *bar*B
343	266	*csu*A/B, *csu*A-C, *csu*E	*pga*A	*aba*I, *aba*R	*plc*C, *plc*D	*bas*A-D, *bas*F-J, *bau*B-F, *ent*E	*ade*G, *ade*H, *bar*A, *bar*B
345	306	*csu*A/B, *csu*A-E	*pga*A-D	*aba*I, *aba*R	*plc*C, *plc*D	*bas*A-D, *bas*F-J, *bau*A-F, *ent*E	*ade*G, *ade*H, *bar*A, *bar*B
349	504	*csu*A/B, *csu*A-E	*pga*A-D	*aba*I, *aba*R	*plc*C, *plc*D	*bas*A-D, *bas*F-J, *bau*A-F, *ent*E	*ade*G, *ade*H, *bar*A, *bar*B
350	509	*csu*A/B, *csu*A-E	*pga*A-D	*aba*I, *aba*R	*plc*C, *plc*D	*bas*A-D, *bas*F-J, *bau*A-F, *ent*E	*ade*G, *ade*H, *bar*A, *bar*B
351	514	*csu*A/B, *csu*A-E	*pga*A-D	*aba*I, *aba*R	*plc*C, *plc*D	*bas*A-D, *bas*F-J, *bau*A-F, *ent*E	*ade*G, *ade*H, *bar*A, *bar*B
352	720	*csu*A/B, *csu*A-E	*pga*A-D	*aba*I, *aba*R	*plc*C, *plc*D	*bas*A-D, *bas*F-J, *bau*A-F, *ent*E	*ade*G, *ade*H, *bar*A, *bar*B

Virulence-associated genes were identified through in silico analysis of whole-genome sequences of *A. baumannii* isolates and were categorized according to their main biological functions. Only genes meeting the sequence identity and coverage thresholds defined by the annotation pipeline were considered present.

The functional categories include: pili/adhesion factors (PA), biofilm formation-associated genes (BF), quorum sensing systems (QS), toxins and hydrolytic enzymes (TE), siderophore-mediated iron acquisition systems (SID), and efflux systems (ESs). These categories reflect major virulence mechanisms involved in host colonization, persistence, iron acquisition, intercellular communication, and antimicrobial resistance-associated efflux activity.

All annotations are based on curated genome-wide in silico predictions, and gene presence reflects detected homologs meeting predefined quality thresholds in the annotation pipeline.

PA, pili/adhesion; BF, biofilm formation; QS, quorum sensing; TE, toxins/enzymes; SID, siderophore-mediated iron acquisition; ESs, efflux systems.

Analysis of genes associated with PA revealed both conserved and variable patterns among the strains. The *csu*A/B gene was detected in all strains. The complete *csu*A–E operon, detected in 75.0% (n = 12) of the strains. The detection of partial operons, such as *csu*A–C and *csu*A–D was observed in fewer strains, as well as the isolated presence of the *csu*E gene in only one strain (6.3%).

Analysis of genes associated with biofilm formation revealed the presence of regulatory genes *bfm*R and *bfm*S, detected in 31.3% (n = 5) of the strains. The *pga*A gene was detected in 100% (n = 16) of the strains. The *pga*B gene was detected in 62.5% (n = 10) of the strains. The *pga*C and *pga*D genes were both detected in 75.0% (n = 12) of the strains.

Assessment of QS genes revealed a broad distribution of *aba*I and *aba*R, with variations among the strains. The *aba*I gene detected in 87.5% (n = 14) of the strains. The *aba*R gene was detected in 100% (n = 16) of the strains. Most strains harbored both genes (*aba*I/*aba*R), however the 130 and 131 strains carried the *aba*R gene only.

The 132 strain harbored the *tle*1 gene, which encodes an effector toxin with lipase activity and is associated with the type VI secretion system, implicated in interbacterial antagonism and potentially in modulating the microenvironment during colonization.

Analysis of genes involved in iron acquisition revealed multiple siderophore systems, which are essential for bacterial survival in environments with limited iron availability. The *bas*A–D operon, detected in 93.8% (n = 15) of the strains. The *bas*F–J genes were also detected in 93.8% (n = 15) of the strains. In contrast, the *bau*A–E genes were detected in only one strain (6.3%; n = 1). Results of all genes observed in all isolates are depicted in **Table 4**.

### 3.5 Inference of clonal structure and genotypic diversity

WGS data were used for in silico analysis of seven housekeeping genes according to both the Oxford (Bartual et al., 2005) and Pasteur (Diancourt et al., 2010) MLST schemes (**Table 5**). The combined use of these schemes was adopted because they provide complementary epidemiological information. The Oxford scheme is generally considered to offer higher discriminatory power due to the greater variability of its target loci, enabling improved differentiation among closely related strains, whereas the Pasteur scheme is more conserved and widely used for global clonal lineage assignment and long-term epidemiological comparisons. Since the two schemes are based on distinct housekeeping genes, discrepancies in sequence type (ST) assignments may occur and are expected in *A. baumannii* population studies. The MLST analysis was performed by detecting and comparing specific allelic profiles within each strain genome, enabling the assignment of their respective sequence types (STs).

**Table 5:** Multilocus sequence typing (MLST) analysis based on Oxford and Pasteur schemes in *A. baumannii* isolates.

Samples	ID	Oxford								Pasteur							
		*glt*A	*gyr*B	*gdh*B	*rec*A	*cpn*60	*gpi*	*rpo*D	STs	*cpn*60	*fus*A	*glt*A	*pyr*G	*rec*A	*rpl*B	*rpo*B	STs
332	010	1	69	90	2	1	79	30	235	3	2	2	2	2	4	8	162
333	012	1	69	90	2	1	79	30	235	3	2	2	2	2	4	8	162
334	027	12	17	12	1	29	108	39	1619	6	6	8	2	3	5	4	15
336	074	01	03	189/3	02	02	102	03	2164/218	2	2	2	2	2	2	2	2
337	075	01	03	189/3	02	02	102	03	2164/218	2	2	2	2	2	2	2	2
338	076	1	69	90	2	1	79	30	235	3	2	2	2	2	4	8	162
339	130	12	15	2	28	29	283	39	3275	6	3	8	4	7	2	4	2526
340	131	21	35	2	28	1	372	4	2459	3	1	7	1	7	2	4	193
341	132	12	15	2	28	29	283	39	3275	6	3	8	4	7	2	4	2526
342	182	01	03	189/3	02	02	102	03	2164/218	2	2	2	2	2	2	2	2
343	266	10	38	182	11	4	98	5	2321	1	1	1	1	5	1	1	1
345	306	1	3	3	2	2	67	3	3214	2	2	2	2	2	2	2	2
349	504	01	03	189/3	02	02	102	03	2164/218	2	2	2	2	2	2	2	2
350	509	10	38	182	11	4	98	5	2321	1	1	1	1	5	1	1	1
351	514	12	15	2	28	29	283	39	3275	6	3	8	4	7	2	4	2526
352	720	01	03	189/3	02	02	102	03	2164/218	2	2	2	2	2	2	38	143

Multilocus sequence typing (MLST) was performed based on two complementary schemes for *A. baumannii*: the Oxford scheme and the Pasteur scheme. The Oxford scheme includes the housekeeping genes *glt*A, *gyr*B, *gdh*B, *rec*A, *cpn*60, *gpi*, and *rpo*D, while the Pasteur scheme is based on *cpn*60, *fus*A, *glt*A, *pyr*G, *rec*A, *rpl*B, and *rpo*B. Sequence types (STs) were assigned according to allele combinations obtained from in silico analysis of whole-genome sequencing data.

Allelic profiles are shown for each locus, and sequence type (ST) designations correspond to the combination of alleles across all seven housekeeping genes within each scheme. Dual values (e.g., 2164/218) indicate closely related or alternative allele assignments differing at a single locus, consistent with microvariation events such as point mutations or recombination within the Oxford scheme. The Oxford scheme provides higher discriminatory power for short-term epidemiological and clonal tracking, whereas the Pasteur scheme reflects broader phylogenetic relationships with greater evolutionary stability.

MLST, multilocus sequence typing; ST, sequence type.

In the Oxford scheme, ST235 predominated in 010, 012, and 076 strains, while ST3275 was observed in 130, 132, and 514 strains. A third relevant cluster included 074, 075, 182, 504, and 720 strains, for which two possible STs (ST2164 and ST218) were assigned. These strains exhibited identical allelic profiles for most loci analyzed, differing only in the *gdh*B gene (189 and 3 alleles). Strains genotyped as ST2321 (266 and 509) exhibited identical allelic profiles across all loci evaluated. Unique STs were also detected, including ST1619 (027 strain), ST2459 (131 strain) and ST3214 (306 strain).

Analysis according to the Pasteur scheme revealed a more conserved and phylogenetically stable clonal structure. Using this scheme, a smaller number of STs (n = 7) were identified than with the Oxford scheme (n = 8). ST2 was the most frequent, detected in 074, 075, 182, 306, and 504 strains. Another distinct clonal cluster was represented by ST2526, detected in 130, 132, and 514 strains. ST1 was identified in 266 and 509 strains, and ST162 in 010, 012, and 076 strains. Less frequent STs were detected, including ST15 (027 strain), ST193 (131 strain), and ST143 (720 strain).

## 4 Discussion

The predominance of *A. baumannii* in respiratory samples from patients with COVID-19 (62.5%) observed in this study is consistent with the widely described epidemiological profile of this nosocomial pathogen, in which the respiratory tract represents the primary site of colonization and infection, particularly among individuals undergoing invasive mechanical ventilation. This finding is especially relevant in the context of COVID-19, given the high frequency of prolonged ventilatory support and the consequent increased susceptibility to ventilator-associated pneumonia. Conversely, the low proportion of isolates obtained from blood samples (6.3%) diverges from data reported in specific clinical populations, in which *A. baumannii* bacteremia shows greater representation, particularly in settings of immunosuppression or intensive exposure to invasive devices (Ayoub Moubareck and Hammoudi Halat, 2020).

Nie et al. (2015) conducted a retrospective study involving solid organ transplant recipients and documented 101 episodes of *A. baumannii* infection in 78 patients, with a predominance of respiratory tract infections (37.6%) and bloodstream infections (35.6%), highlighting the high frequency of bacteremia in this immunosuppressed context. These data support the notion that the distribution of isolation sites can vary substantially depending on cohort characteristics, the degree of immunosuppression, exposure to invasive procedures, and inclusion criteria (Nie et al., 2015).

MALDI-TOF MS identification in the present study fell within the range accepted for reliable species-level identification, as more than two-thirds of the strains exhibited scores ≥ 2.11. The high proportion of scores classified as consistency A indicates highly reliable identifications, whereas scores classified as consistency B may reflect limitations of the spectral library or intrinsic proteomic characteristics of the analyzed strains. Methodological studies confirm that MALDI-TOF MS performance is strongly dependent on the quality and comprehensiveness of the reference database (Croxatto et al., 2012).

In this context, Jeong et al. (2016) evaluated the Bruker MALDI-TOF MS system for the identification of 517 clinical *Acinetobacter* strains and observed an initial concordance of 69.8% with *rpo*B gene sequencing (β subunit of RNA polymerase). Following the incorporation of additional profiles and the use of an expanded spectral library, concordance increased to 100%, demonstrating the critical impact of library optimization on diagnostic accuracy (Jeong et al., 2016).

The analyzed genomes, with lengths ranging from 3, 822, 253 to 4, 256, 491 bp, fall within the typical range reported for complete *A. baumannii* genomes. Records referenced from NCBI RefSeq indicate genome sizes predominantly between ~3.8 Mb (ASM250414v1) and ~4.2 Mb (ASM326427v1), confirming that the obtained assemblies are consistent with the expected biological variability for the species (NCBI RefSeq, 2025). The greater similarity observed between the clinical strains and the AB5075-UW strain suggests that the evolutionary mechanisms responsible for the adaptation of *A. baumannii* to the hospital environment, particularly the acquisition of resistance genes and other accessory elements through horizontal gene transfer, may have played an important role in shaping these genomes (Muthuraman et al., 2026). The conservation of regions associated with antimicrobial resistance relative to AB5075-UW, together with the divergence observed when compared to ATCC 17978, reinforces the notion that contemporary clinical lineages share a set of adaptive genetic features that are absent or less represented in older reference strains (Gallagher et al., 2015). This pattern has been consistently reported in comparative genomic studies of *A. baumannii* and reflects the remarkable ability of the species to remodel its genome in response to the selective pressures present in hospital environments, including the accumulation of mobile genetic elements, insertion sequences, resistance islands, and virulence-associated determinants (Bian et al., 2021; Wareth et al., 2021a).

Completeness values ranged from 97.4% to 100%, with most strains exhibiting ≥ 99.5%, and contamination levels between 0% and 0.63%. These parameters are consistent with high-quality public genomes. Pearl and Anbarasu (2025), in a large-scale genomic analysis based exclusively on *A. baumannii* genomes from NCBI, consistently reported completeness above 99% and contamination below 1%, using BUSCO, QUAST, and CheckM as quality control metrics. These results support the suitability of the genomes in the present study for phylogenomic, pangenomic, and functional analyses (Pearl and Anbarasu, 2025).

The genomic variability observed among the strains (3.82–4.26 Mb) reflects the pan-genome dynamics previously described for *A. baumannii*. Karampatakis et al. (2024) demonstrated that the species possesses an open pan-genome and high genomic plasticity, with a broad diversity of accessory genes driven by the acquisition of mobile genetic elements (genomic islands, plasmids, integrons, and insertion sequences). This phenomenon is closely associated with hospital adaptation, the emergence of high-risk lineages, and the dissemination of resistance and virulence determinants (Karampatakis et al., 2024).

The high prevalence of MDR and CRAB isolates (93.8% each) observed in the present study highlights an alarming resistance profile. Stoian et al. (2025) reported MDR rates frequently exceeding 70–90% in hospital cohorts, accompanied by particularly high levels of carbapenem resistance, emphasizing the need for rigorous microbiological surveillance (Stoian et al., 2025). Similarly, Ababneh et al. (2025) found that approximately 90% of *A. baumannii* strains recovered from ICUs in Jordan exhibited extensively drug-resistant (XDR) phenotypes and predominantly carried *blaOXA-23-like* genes located on mobile genetic elements, facilitating the dissemination of resistance to IPM and MEM (Ababneh et al., 2025). Furthermore, Asmare et al. (2025), in a meta-analysis, reported substantial regional variability in antimicrobial resistance patterns, with resistance to IPM and MEM ranging approximately from 33.5% to 43.6%, TZP resistance frequently exceeding 60%, and MDR prevalence ranging from 88% to 93% across different settings, reflecting both geographic and methodological heterogeneity (Asmare et al., 2025).

These findings are consistent with the resistance profile observed in the present study, in which resistance to TZP, CAZ, and IPM reached 93.8%, whereas resistance to MEM was observed in 87.5% of the isolates. These values approach, and in some cases exceed, the upper limits reported in healthcare settings exposed to intense antimicrobial selective pressure. In contrast, resistance to AMP/SUL was detected in only 6.3% of the isolates, indicating relatively preserved susceptibility to this antimicrobial combination and supporting observations reported in previous studies.

In-silico analysis of the WGS data from *A. baumannii* revealed a broad and heterogeneous repertoire of genetic determinants of resistance, with marked variations across different antimicrobial classes. This pattern reflects the well-recognized genomic plasticity of this pathogen, which has historically exhibited a high adaptive capacity (Woo et al., 2002; Rodrigues et al., 2024), and is supported by recent genomic studies demonstrating the continuous acquisition of resistance genes mediated by mobile genetic elements in clinical MDR strains (Jouybari et al., 2021; Rashvand et al., 2021).

The observed profile of genes encoding aminoglycoside-modifying enzymes (AMEs), including AAC, adenyltransferases/nucleotidyltransferases (AAD/ANT), APHs, and ARM/RMT, with high frequencies of *aad*A1, *aph*(3′)-Ia, *aph*(3″)-Ib, and *aph*(6)-Id, as well as the detection of *arm*A and *rmt*B in some strains, is consistent with previously reported genomic data for *A. baumannii*. Jouybari et al. (2021), analyzing 100 clinical strains, reported high prevalences of *aph*A6 (77%), *aad*B (73%), and *aad*A1 (33%), associated with elevated levels of resistance to gentamicin, tobramycin, and AMK (Jouybari et al., 2021). Similarly, Rashvand et al. (2021), in an Iranian cohort of 192 clinical strains, observed gentamicin resistance in 98.4% and AMK resistance in 83.9% of strains, with frequent detection of AME genes, including *aph*(3′)-VI (59.3%), *aac*(6′)-Ib and *aac*(3)-II (39.2% each), and *aph*(3′)-Ia (31.7%), in addition to the 16S rRNA methylase armA in 69.8% of strains. These findings highlight the coexistence of multiple aminoglycoside resistance mechanisms in clinical contexts under high selective pressure, particularly during the COVID-19 pandemic (Rashvand et al., 2021).

The high prevalence of genes encoding oxacillinases (*bla*OXA-23, *bla*OXA-51, *bla*OXA-66, and *bla*OXA-69), together with the presence of AmpC/ESBL, aligns with global resistance patterns in *A. baumannii* (Sahayarayan et al., 2024). Wang et al. (2018), in a large clinical cohort in Taiwan, reported that *bla*OXA-23-like was detected in 88.1% of carbapenem-resistant strains, substantially more frequent than *bla*OXA-24-like (4.1%) and *bla*OXA-58-like (0%), while *bla*OXA-51-like was present in all strains but showed no consistent correlation with resistance in the absence of the ISAba1 insertion sequence (Wang et al., 2018). These findings reinforce that *bla*OXA-23-like constitutes a major genetic determinant of IPM and MEM resistance, frequently associated with mobile elements that facilitate both horizontal gene transfer and gene overexpression (Wang et al., 2018).

In Brazil, Camargo et al. (2022) reported an outbreak involving *A. baumannii* International Clone 2 (IC-2) during the first wave of the pandemic, in which 76% of colonized or infected patients were associated with a single OXA-23-producing clone. All strains exhibited high-level resistance to aminoglycosides, fluoroquinolones, and β-lactams (>95%), in addition to carrying the *Arm*A methylase, contributing to extreme aminoglycoside resistance (Camargo et al., 2022). Similar findings were reported in retrospective ICU studies involving COVID-19 patients, in which all carbapenem-resistant strains harbored *bla*OXA-23 and belonged predominantly to ST2, displaying universal resistance to IPM (Dobrović et al., 2023).

The low detection of β-lactamases (*bla*TEM-1B, *bla*ADC-25, and *bla*ADC-25B), in contrast to the high phenotypic resistance to TZP and CAZ (93.8% each) and the low resistance to ampicillin–sulbactam (6.3%), suggests a predominant contribution of class D oxacillinases and possibly AmpC overexpression mediated by insertion elements. Han et al. (2017) reported an extremely high prevalence of *bla*OXA-23-like (~97.8%) and *bla*TEM-1 (~91.1%), while *bla*OXA-51-like was present in all analyzed strains without direct correlation to resistance in the absence of ISAba1, reinforcing the evolutionary predominance of oxacillinases in carbapenem and third-generation cephalosporin resistance (Han et al., 2017).

Additional resistance determinants were also detected, including *erm*(42), associated with the MLS phenotype via 23S rRNA methylation, and *tet*(B), found in 62.5% of the strains, consistent with studies describing it as the main determinant of tetracycline resistance mediated by ESs (Fyfe et al., 2016; Khan et al., 2024). Khan et al. (2024), in an analysis of 82 clinical *A. baumannii* from Bangladesh, reported *tet*(B) as the most prevalent gene (46.3%) among the strains, associated with tetracycline resistance and frequently coexisting with other multidrug-resistance determinants (Khan et al., 2024).

Regarding phenicols, the concurrent detection of *flo*R and *cat*B8 suggests the combined action of efflux and enzymatic inactivation mechanisms. *flo*R encodes a major facilitator superfamily (MFS, major facilitator superfamily) efflux protein capable of actively exporting CHL/florfenicol out of the cell, whereas *cat*B8 encodes an acetyltransferase that chemically inactivates the antimicrobial via acetylation. Wareth et al. (2021b) demonstrated the presence of these genes in carbapenem-resistant clinical genomes from Southeast Asia, highlighting the coexistence of multiple determinants of CHL/florfenicol resistance in some strains; *cat*B8 was the most frequent (~13%), while *flo*R was present in a smaller subset, indicating the simultaneous occurrence of enzymatic inactivation and efflux mechanisms in specific genomes (Wareth et al., 2021b).

The high prevalence of sul1 and *sul*2 (50–56%) aligns with genomic data indicating widespread dissemination of these determinants in *A. baumannii*. These genes encode sulfonamide-insensitive variants of dihydropteroate synthase and are frequently detected together, reflecting broadly distributed multidrug-resistance profiles in clinical populations (Khan et al., 2024). In clinical *A. baumannii* genomes isolated in Bangladesh, Khan et al. (2024) observed high proportions of sul1 and *sul*2, associated with extensive repertoires of resistance genes and MDR phenotypes. These findings support that, even in strains with variable genetic content, *sul*1 and *sul*2 remain among the main determinants of sulfonamide resistance in *A. baumannii*, consistent with the profile observed in the present study (Khan et al., 2024).

The in-silico analysis of WGS data from *A. baumannii* revealed a heterogeneous repertoire of virulence genes associated with adhesion, biofilm formation, QS, iron acquisition, and ESs.

In the present study, the *csu*A/B gene was detected in 100% of the strains, while the complete *csu*A–E operon was present in 75%. This profile indicates that most strains possess the genetic machinery required for the assembly of chaperone–usher type fimbriae, which are recognized as mediators of initial adhesion to biotic and abiotic surfaces and of biofilm formation in *A. baumannii* (Tomaras et al., 2003).

In clinical IC-2 strains, Kishii et al. (2020) reported the presence of the complete *csu*A–E operon in 73.7% of strains, which was associated with enhanced biofilm-forming capacity in crystal violet assays. The proximity of this value to that observed in the present study (75%) suggests a high level of conservation of this operon in clinical populations. However, Kishii et al. (2020) also demonstrated that not all IC-2 strains carry the complete operon, supporting the heterogeneity observed here, including partial profiles (*csu*A–C, *csu*A–D) and the isolated detection of *csu*E (6.3%), which may modulate adhesion efficiency and strain-specific adaptive strategies (Kishii et al., 2020).

The detection of *omp*A gene in only 37.5% of the strains contrasts with the 97.7% prevalence reported by Shaikh et al. (2025) in 44 analyzed strains. This discrepancy may reflect local genomic diversity or methodological differences. Considering the multifunctional role of OmpA in adhesion, invasion, and biofilm formation, the low frequency observed here suggests the potential involvement of compensatory mechanisms mediated by other surface proteins (Shaikh et al., 2025).

The genes *pga*A, *pga*B, *pga*C, and *pga*D were detected in 100%, 62.5%, 75%, and 75% of the strains, respectively, indicating interstrain variability in the synthesis and export of poly-β-1, 6-N-acetylglucosamine (PNAG). Complete deletion of the *pga*ABCD operon results in loss of PNAG production and biofilm formation, with phenotypic restoration upon genetic complementation (Choi et al., 2009). In contrast, Supreeta et al. (2025) reported prevalences of *pga*B (93%) and *pga*C (89.7%), suggesting geographic or population-specific variations. Functionally, each gene of the operon contributes integrally to biofilm density and stability, with the absence of any component significantly impairing this phenotype; for example, *pga*A encodes the protein responsible for PNAG transport to the extracellular space, *pga*B promotes cell–cell adhesion and matrix stabilization, *pga*C catalyzes polysaccharide polymerization, and *pga*D facilitates efficient assembly of the secretion system, ensuring proper PNAG deposition within the biofilm matrix (Supreeta et al., 2025).

The core QS genes, *aba*I and *aba*R, were detected in 87.5% and 100% of the strains, respectively. The AbaI/AbaR system, analogous to LuxI/LuxR, regulates biofilm formation and the expression of virulence factors (Xiong et al., 2022). The *aba*I gene encodes an autoinducer synthase responsible for the production of N-acyl homoserine lactones (AHLs), whereas *aba*R encodes the transcriptional receptor that senses these signals and activates cell-density-dependent regulatory cascades. Tang et al. (2020) reported the coexistence of both genes in 76.25% of clinical strains evaluated, with individual frequencies of 83.75% for *aba*I and 78.75% for *aba*R in strains from China; however, they observed variable AHL production, highlighting a disconnect between genetic presence and QS system functionality (Tang et al., 2020). The absence of *aba*I in two strains from the present study may reflect reliance on exogenous signals (“cross-feeding”), a phenomenon described by Sun et al. (2021), in which QS receptors remain functional despite the loss of signal synthases. The universal conservation of *aba*R suggests an adaptive advantage in multispecies clinical environments (Sun et al., 2021).

Genes related to iron acquisition (*bas*/*bau*), including *bas*A–D and *bau*A–E, were widely detected in the strains analyzed, consistent with the high conservation of this gene cluster in clinical *A. baumannii* Conde-Pérez et al., 2021). The *bas*/*bau* locus encodes the biosynthetic enzymes (*bas*A–J) and transport components (*bau*A–E) of acinetobactin, with expression regulated by iron availability, a micronutrient whose accessibility is tightly restricted by the host during infection (Artuso et al., 2023).

In comprehensive genomic studies, the *bas*/*bau* cluster has been shown to be highly conserved in clinical *A. baumannii*, with nearly all analyzed genomes containing the full complement of genes; only a small fraction (~1.2%) lacked essential genes such as *ent*A, required for the synthesis of the 2, 3-dihydroxybenzoate (DHBA) precursor, a key intermediate in siderophore biosynthesis (Artuso et al., 2023). Additional epidemiological evidence supports the high frequency of genes involved in acinetobactin biosynthesis, with approximately 99% prevalence of *bas*D in a cohort of 100 MDR strains, highlighting that the ability to synthesize acinetobactin is a ubiquitous and central determinant of this pathogen’s virulence (Depka et al., 2023). These findings are consistent with functional data demonstrating that acinetobactin is the most critical siderophore for *A. baumannii* survival under iron-restricted conditions both *in vitro* and *in vivo* (Sheldon and Skaar, 2020).

The detection of *ent*E gene in some strains suggests the presence of auxiliary siderophore pathways, potentially contributing to the production of metabolites involved in iron acquisition. This finding is consistent with the diversity of siderophore systems described in *A. baumannii*, which includes acinetobactin, baumannoferrins, and fimsbactins (Sheldon and Skaar, 2020). In contrast, the detection of *iro*E gene in only a single strain indicates that less conserved siderophore systems, possibly acquired via horizontal gene transfer, are restricted to specific subgroups and do not exhibit broad population-level distribution, in agreement with genomic data showing low frequencies of additional siderophore loci in large cohorts of clinical strains (Artuso et al., 2023).

The genes *ade*F, *ade*G, and *ade*H encode, respectively, the periplasmic adaptor protein, the inner membrane transporter, and the outer membrane channel of the RND-ESs (AdeFGH), recognized as one of the main determinants of antimicrobial resistance in *A. baumannii* due to its ability to export multiple classes of toxic compounds (Ajoseh et al., 2025). In the present study, these genes were detected in different combinations across the strains, concurrently with the near-universal presence of the regulatory *bar*A/*bar*B genes (absent only in 012 strain), indicating heterogeneity both in ES composition and in their regulatory mechanisms.

In general, RND-ESs represent a major cornerstone of the MDR phenotype in *A. baumannii*, due to their broad substrate specificity, which includes fluoroquinolones, tetracyclines, β-lactams, macrolides, as well as detergents and biocides. By lowering the intracellular concentration of these compounds, these systems contribute to elevated minimum inhibitory concentrations (MICs) in clinical MDR strains (Coyne et al., 2011).

Molecular studies indicate that, in addition to the AdeABC system, the AdeFGH and AdeIJK systems are widely distributed in clinical strains, although their relative contribution to the resistance profile varies depending on the epidemiological context and the panel of antimicrobials tested. Notably, AdeFGH exhibits high prevalence in clinical cohorts, with frequencies exceeding 90% for *ade*G gene, and is associated with resistance to fluoroquinolones and other classes, as well as a positive correlation between the presence of *ade*F/*ade*G and elevated resistance levels to ciprofloxacin and norfloxacin (Kaviani et al., 2020).

The regulation of the AdeFGH system involves the *bar*A/*bar*B regulators, which modulate the expression of structural genes in response to environmental signals and antimicrobial stress. This regulatory axis is distinct from the AdeRS system, which controls the AdeABC operon. Although less well characterized, recent evidence indicates that *bar*A/*bar*B influence the expression dynamics of AdeFGH throughout growth phases and under varying selective pressures, including exposure to antimicrobials and toxic compounds (Wimalasekara et al., 2025).

The combinatorial variability of *ade*F, *ade*G, and *ade*H observed among the strains suggests the coexistence of different functional architectures of RND-ESs, reflecting regulatory plasticity and metabolic adaptability. Comparative studies have shown that the presence of multiple RND systems does not necessarily imply simultaneous overexpression or a direct linear relationship with resistance levels, but rather a flexible response to heterogeneous environmental pressures (Abd El-Rahman et al., 2023).

In addition to AdeFGH, the AdeABC and AdeIJK systems are frequently detected in clinical strains and contribute not only to antimicrobial resistance but also to adaptation-associated phenotypes, including biofilm formation and cellular invasion. The AdeIJK system, in particular, is considered ancestral and broadly conserved within the genus *Acinetobacter*, providing a baseline resistance that can be enhanced through the acquisition or activation of additional systems, such as AdeFGH (Darby et al., 2023).

Collectively, the widespread detection of *bar*A/*bar*B in the present cohort, except in 012 strain, underscores that ESs regulation remains functional in the majority of strains, enabling adaptive modulation of resistance in response to diverse environmental and pharmacological conditions (Wimalasekara et al., 2025).

Data available in PubMLST (https://pubmlst.org/) indicate that thousands of STs have been described for *A. baumannii*, with over 20, 000 strains typed as of 2026 using the Oxford and Pasteur MLST schemes. This high number reflects the remarkable genetic plasticity of the species and its ongoing capacity for diversification, extensively documented in global population studies.

Analysis of the seven housekeeping genes using the Oxford and Pasteur MLST schemes revealed, within our set of strains, the coexistence of widely disseminated clones alongside unique STs. Under the Oxford scheme, ST235 and ST3275 were the most prevalent, in addition to closely related clusters between ST2164 and ST218, which differed only in the *gdh*B allele. The detection of rare STs, such as ST2321, ST1619, ST2459, and ST3214, underscores the occurrence of local microdiversification within the studied population. In contrast, analysis using the Pasteur scheme showed a more conserved distribution, with predominance of ST2 and the presence of less frequent STs (ST2526, ST162, ST1, ST15, ST193, and ST143), reflecting the greater phylogenetic stability characteristic of this scheme.

Although both MLST schemes analyze seven housekeeping genes, they differ substantially in locus selection. The Oxford scheme includes genes such as *gdh*B, *gpi*, and *gyr*B, which are known to be more prone to recombination and allelic variability. In particular, the *gpi* gene, involved in central metabolic pathways, exhibits a high rate of homologous recombination, contributing to the generation of new allelic profiles. In contrast, the loci selected by the Pasteur scheme display lower variability among phylogenetically related strains, providing greater clonal stability for population inferences (Mateo Estrada and Castillo Ramírez, 2026).

Comparative studies consistently demonstrate that the Oxford scheme discriminates a greater number of STs from the same set of strains compared to the Pasteur scheme. Hua et al. (2020), in a study involving 166 A*. baumannii* strains, assigned 145 strains to 11 distinct STs using the Oxford scheme, whereas the Pasteur scheme classified all these strains into a single ST, highlighting Oxford’s higher sensitivity for detecting microevolutionary variations that remain grouped under Pasteur (Hua et al., 2020). Consequently, strains classified as ST2 by the Pasteur scheme, frequently associated with IC-2, can be subdivided into multiple STs by the Oxford scheme, such as ST208, ST425, ST451, and ST436. This subdivision results from Oxford’s greater sensitivity to microvariations in recombinogenic loci, enabling a more detailed analysis of intraclone diversity within high-risk clones (Gaiarsa et al., 2019).

However, the high resolution of the Oxford scheme can be influenced by recombination events and the presence of paralogous loci, such as the occurrence of a second copy of the *gdh*B gene. These features may generate additional allelic profiles and new STs that do not necessarily reflect true population differences. In contrast, the Pasteur scheme tends to group strains into phylogenetically more conserved STs, providing a more stable overview of global clonal structure, particularly suitable for international comparisons, detection of widely recognized clones (IC-1, IC-2, and IC-3), and robust population analyses (Gaiarsa et al., 2019; Mateo Estrada and Castillo Ramírez, 2026).

During the COVID-19 pandemic, MLST-based epidemiological studies reported complex patterns of clonal diversification of *A. baumannii* in ICUs, characterized by the coexistence of multiple STs and temporal variations in clone prevalence. In a hospital in northern China, 95 clinical strains collected between January 2020 and December 2022 were assigned to 28 STs using the Oxford scheme, with predominance of ST540 and ST469 (13.7% each), followed by ST373 (8.4%), ST938 (7.4%), and ST208 (6.3%). At the national level, ST208 was the most widely disseminated (22.1%). Using the Pasteur scheme, there was a marked predominance of ST2 (58.9%), followed by ST40 (9.5%) and ST33 (4.2%), highlighting the persistence and global stability of ST2 during the pandemic period (Huang et al., 2024).

These findings are consistent with the results of the present study, in which ST2 (Pasteur) was the predominant type, indicating that this phylogenetically stable clone remains widely disseminated. Simultaneously, the detection of multiple STs using the Oxford scheme, including less frequent and seemingly local variants, reflects the adaptive capacity and ongoing microdiversification of *A. baumannii* in hospital environments subjected to intense antimicrobial pressure during the pandemic in Brazil (Pearl and Anbarasu, 2025).

In addition to the predominant STs, the rare STs identified in this study (ST218, ST2321, ST1619, ST2459, ST3214, and ST143) reflect a pattern of microevolutionary diversity widely reported in MLST studies conducted across different hospital settings. While some STs form widely distributed clonal complexes, such as CC92/IC-2, many occur at low frequency or are unique to specific cohorts, arising through point recombination, local variants, or inter-hospital transfer (Choudhary and Shariff, 2025; Zueter et al., 2025).

ST218, for instance, has been previously reported in European cohorts, frequently associated with MDR or XDR phenotypes and resistance to β-lactams and carbapenems, highlighting microvariations within widely disseminated clonal complexes. Unique STs, such as ST2321 and ST1619, although sparsely documented in large cohorts, reflect a recurrent pattern in molecular surveillance, in which novel STs or exclusive variants are assigned to individual clinical strains deposited in global MLST databases. In studies reporting previously undescribed STs, such as ST3281, ST3282, and ST3285, these types emerged within the same hospital environment without evidence of significant clonal expansion, suggesting the presence of local reservoirs or isolated events of gene recombination (Sidor-Dzitkowska et al., 2025).

Similarly, STs such as ST2459 and ST3214 fit this pattern of low-frequency clonal microvariations. Although infrequently reported, their detection in larger cohorts indicates that *A. baumannii* harbors a broad genetic pool, within which low-frequency variants can emerge and disappear in response to local selective pressures, clinical practices, and recombination dynamics among housekeeping loci (Pan et al., 2025). ST143, whose allelic profile differs primarily at the *rpo*B locus, exemplifies a point event of genetic diversification within a phylogenetically stable clone, resulting in a novel ST without substantial impact on the overall phylogeny.

Taken together, the simultaneous occurrence of widely disseminated STs (such as IC-2/ST2 and ST208) and rare or unique STs (ST218, ST2321, ST1619, ST2459, ST3214, and ST143) demonstrates that the *A. baumannii* population is highly dynamic and evolutionarily flexible. This population structure is shaped by local selective pressures, hospital transmission dynamics, and intensive antibiotic use, underscoring the need for continuous epidemiological surveillance to detect both broadly disseminated clones and rare local variants, particularly in settings of high selective pressure, such as those observed during the COVID-19 pandemic (Ajoseh et al., 2025; Li et al., 2025; Sidor-Dzitkowska et al., 2025).

## 5 Conclusion

The present study demonstrates that clinical *A. baumannii* strains isolated during the COVID-19 pandemic in Brazil exhibit high genomic diversity and a broad spectrum of resistance to multiple antimicrobial classes, with a high prevalence of MDR and CRAB phenotypes. Genomic characterization revealed multiple determinants associated with resistance to aminoglycosides (AGs), β-lactams, macrolides, tetracyclines (TCs), chloramphenicol (CHL), and sulfonamides, highlighting the limited therapeutic options available. In addition, virulence genes related to cell adhesion, biofilm formation, quorum sensing (QS), iron acquisition, and efflux systems (ESs) were identified, emphasizing their role in the persistence, adaptation, and nosocomial dissemination of *A. baumannii*.

These findings reinforce the widespread dissemination of MDR/CRAB lineages during the pandemic and underscore the importance of continuous genomic surveillance, particularly in hospital environments subjected to intense antimicrobial selective pressure. They also emphasize the need for integrated infection prevention and control strategies combined with rational antimicrobial use as essential measures to contain the spread of high-risk clones and reduce the impact of these strains in healthcare settings. In this context, the implementation of routine genomic surveillance programs in Brazilian hospitals, integrated with clinical microbiology laboratories and infection control committees, may contribute to the early detection of emerging high-risk clones, monitoring of resistance dissemination, and optimization of outbreak prevention and antimicrobial stewardship strategies.

## Data Availability

The datasets presented in this study can be found in online repositories. The names of the repository/repositories and accession number(s) can be found in the article/supplementary material.

## References

[B1] Ababneh Q. Aldaken N. Jaradat Z. Al-Rousan E. Inaya Z. Alsaleh D. . (2025). Predominance of extensively-drug resistant Acinetobacter baumannii carrying bla OXA-23 in Jordanian patients admitted to the intensive care units. PloS One 20, e0317798. doi: 10.1371/journal.pone.0317798 40014590 PMC11867332

[B2] Abd El-Rahman O. A. Rasslan F. Hassan S. S. Ashour H. M. Wasfi R. (2023). The RND efflux pump gene expression in the biofilm formation of Acinetobacter baumannii. Antibiotics (Basel) 12, 419. doi: 10.3390/antibiotics12020419 36830328 PMC9952185

[B3] Ahuatzin-Flores O. E. Torres E. Chávez-Bravo E. (2024). Acinetobacter baumannii, a multidrug-resistant opportunistic pathogen in new habitats: a systematic review. Microorganisms 12, 644. doi: 10.3390/microorganisms12040644 38674589 PMC11051781

[B4] Ajoseh S. O. Anjorin A. A. Salami W. O. Brangsch H. Neubauer H. Wareth G. . (2025). Comprehensive molecular epidemiology of Acinetobacter baumannii from diverse sources in Nigeria. BMC Microbiol. 25, 178. doi: 10.1186/s12866-025-03917-5 40165088 PMC11956268

[B5] Alcock B. P. Huynh W. Chalil R. Smith K. W. Raphenya A. R. Wlodarski M. A. . (2023). CARD 2023: expanded curation, support for machine learning, and resistome prediction at the Comprehensive Antibiotic Resistance Database. Nucleic Acids Res. 51, D690–D699. doi: 10.1093/nar/gkac920 36263822 PMC9825576

[B6] Artuso I. Poddar H. Evans B. A. Visca P. (2023). Genomics of Acinetobacter baumannii iron uptake. Microb. Genom. 9, mgen001080. doi: 10.1099/mgen.0.001080 37549061 PMC10483418

[B7] Asmare Z. Tamrat E. Erkihun M. Endalamaw K. Alelign D. Getie M. . (2025). Antimicrobial resistance pattern of Acinetobacter baumannii clinical isolates in Ethiopia: a systematic review and meta-analysis. BMC Infect. Dis. 25, 518. doi: 10.1186/s12879-025-10923-5 40221655 PMC11994026

[B8] Ayoub Moubareck C. Hammoudi Halat D. (2020). Insights into Acinetobacter baumannii: a review of microbiological, virulence, and resistance traits in a threatening nosocomial pathogen. Antibiotics (Basel) 9, 119. doi: 10.3390/antibiotics9030119 32178356 PMC7148516

[B9] Bartual S. G. Seifert H. Hippler C. Luzon M. A. Wisplinghoff H. Rodríguez-Valera F. (2005). Development of a multilocus sequence typing scheme for characterization of clinical isolates of Acinetobacter baumannii. J. Clin. Microbiol. 43, 4382–4390. doi: 10.1128/JCM.43.9.4382-4390.2005 16145081 PMC1234098

[B10] Bian X. Liu X. Zhang X. Li X. Zhang J. Zheng H. . (2021). Epidemiological and genomic characteristics of Acinetobacter baumannii from different infection sites using comparative genomics. BMC Genomics 22, 530. doi: 10.1186/s12864-021-07842-5 34247587 PMC8272988

[B11] Camargo C. H. Yamada A. Y. Nagamori F. O. de Souza A. R. Tiba-Casas M. R. de Moraes França F. A. . (2022). Clonal spread of ArmA- and OXA-23-coproducing Acinetobacter baumannii International Clone 2 in Brazil during the first wave of the COVID-19 pandemic. J. Med. Microbiol. 71 (4). doi: 10.1099/jmm.0.001509 35417321

[B12] Carvalheira A. Silva J. Teixeira P. (2021). Acinetobacter spp. in food and drinking water: a review. Food Microbiol. 95, 103675. doi: 10.1016/j.fm.2020.103675 33397609

[B13] Choi A. H. Slamti L. Avci F. Y. Pier G. B. Maira-Litrán T. (2009). The pgaABCD locus of Acinetobacter baumannii encodes the production of poly-beta-1-6-N-acetylglucosamine, which is critical for biofilm formation. J. Bacteriol. 191, 5953–5963. doi: 10.1128/JB.00647-09 19633088 PMC2747904

[B14] Choudhary J. Shariff M. (2025). Multilocus sequence typing of clinical and colonizing isolates of Acinetobacter baumannii and comparison with world isolates. BMC Microbiol. 25, 196. doi: 10.1186/s12866-025-03804-z 40186116 PMC11969935

[B15] Clinical and Laboratory Standards Institute (2023). Performance standards for antimicrobial susceptibility testing.

[B16] Conde-Pérez K. Vázquez-Ucha J. C. Álvarez-Fraga L. Ageitos L. Rumbo-Feal S. Martínez-Guitián M. . (2021). In-depth analysis of the role of the acinetobactin cluster in the virulence of Acinetobacter baumannii. Front. Microbiol. 12, 752070. doi: 10.3389/fmicb.2021.752070 34675911 PMC8524058

[B17] Coyne S. Courvalin P. Périchon B. (2011). Efflux-mediated antibiotic resistance in Acinetobacter spp. Antimicrob. Agents Chemother. 55, 947–953. doi: 10.1128/AAC.01388-10 21173183 PMC3067115

[B18] Croxatto A. Prod'hom G. Greub G. (2012). Applications of MALDI-TOF mass spectrometry in clinical diagnostic microbiology. FEMS Microbiol. Rev. 36, 380–407. doi: 10.1111/j.1574-6976.2011.00298.x 22092265

[B19] Darby E. M. Bavro V. N. Dunn S. McNally A. Blair J. M. A. (2023). RND pumps across the genus Acinetobacter: AdeIJK is the universal efflux pump. Microb. Genom. 9, mgen000964. doi: 10.1099/mgen.0.000964 36995182 PMC10132057

[B20] Depka D. Bogiel T. Rzepka M. Gospodarek-Komkowska E. (2023). The prevalence of virulence factor genes among carbapenem-non-susceptible Acinetobacter baumannii clinical strains and their usefulness as potential molecular biomarkers of infection. Diagnostics (Basel) 13, 1036. doi: 10.3390/diagnostics13061036 36980344 PMC10047099

[B21] Diancourt L. Passet V. Nemec A. Dijkshoorn L. Brisse S. (2010). The population structure of Acinetobacter baumannii: expanding multiresistant clones from an ancestral susceptible genetic pool. PloS One 5, e10034. doi: 10.1371/journal.pone.0010034 20383326 PMC2850921

[B22] Dobrović K. Škrobo T. Selec K. Jelić M. Čivljak R. Peršec J. . (2023). Healthcare-associated bloodstream infections due to multidrug-resistant Acinetobacter baumannii in COVID-19 intensive care unit: a single-center retrospective study. Microorganisms 11, 774. doi: 10.3390/microorganisms11030774 36985347 PMC10056625

[B23] França D. A. Nascimento G. V. Silva K. M. R. Pinheiro I. M. Carneiro M. O. Marques M. C. A. . (2025). Study of prevalence and sensitivity profile of Acinetobacter baumannii infections in health units in the municipality of Teresina, PI, Brazil. Rev. Cereus 17, 126–138. doi: 10.18605/2175-7275/cereus.v17n3p126-138

[B24] Fyfe C. Grossman T. H. Kerstein K. Sutcliffe J. (2016). Resistance to macrolide antibiotics in public health pathogens. Cold Spring Harb. Perspect. Med. 6, a025395. doi: 10.1101/cshperspect.a025395 27527699 PMC5046686

[B25] Gaiarsa S. Batisti Biffignandi G. Esposito E. P. Castelli M. Jolley K. A. Brisse S. . (2019). Comparative analysis of the two Acinetobacter baumannii multilocus sequence typing (MLST) schemes. Front. Microbiol. 10, 930. doi: 10.3389/fmicb.2019.00930 31130931 PMC6510311

[B26] Gallagher L. A. Ramage E. Weiss E. J. Radey M. Hayden H. S. Held K. G. . (2015). Resources for genetic and genomic analysis of emerging pathogen Acinetobacter baumannii. J. Bacteriol. 197, 2027–2035. doi: 10.1128/JB.00131-15 25845845 PMC4438207

[B27] Gheorghe-Barbu I. Dragomir R. I. Gradisteanu Pircalabioru G. Surleac M. Dinu I. A. Gaboreanu M. D. . (2024). Tracing Acinetobacter baumannii's journey from hospitals to aquatic ecosystems. Microorganisms 12, 1703. doi: 10.3390/microorganisms12081703 39203545 PMC11356923

[B28] Grant J. R. Enns E. Marinier E. Mandal A. Herman E. K. Chen C. . (2023). Proksee: in-depth characterization and visualization of bacterial genomes. Nucleic Acids Res. 51, W484–W492. doi: 10.1093/nar/gkad326 37140037 PMC10320063

[B29] Han L. Lei J. Xu J. Han S. (2017). blaOXA-23-like and blaTEM rather than blaOXA-51-like contributed to a high level of carbapenem resistance in Acinetobacter baumannii strains from a teaching hospital in Xi'an, China. Med. (Baltimore) 96, e8965. doi: 10.1097/MD.0000000000008965 29310399 PMC5728800

[B30] Hua X. Zhang L. He J. Leptihn S. Yu Y. (2020). Population biology and epidemiological studies of Acinetobacter baumannii in the era of whole genome sequencing: is the Oxford scheme still appropriate? Front. Microbiol. 11, 775. doi: 10.3389/fmicb.2020.00775 32411113 PMC7201049

[B31] Huang X. Ning N. Li D. Chen S. Zhang L. Wang H. . (2024). Molecular epidemiology of Acinetobacter baumannii during COVID-19 at a hospital in northern China. Ann. Clin. Microbiol. Antimicrob. 23, 63. doi: 10.1186/s12941-024-00716-0 39026334 PMC11264759

[B32] Jeong S. Hong J. S. Kim J. O. Kim K. H. Lee W. Bae I. K. . (2016). Identification of Acinetobacter species using matrix-assisted laser desorption ionization-time of flight mass spectrometry. Ann. Lab. Med. 36, 325–334. doi: 10.3343/alm.2016.36.4.325 27139605 PMC4855052

[B33] Jouybari M. A. Ahanjan M. Mirzaei B. Goli H. R. (2021). Role of aminoglycoside-modifying enzymes and 16S rRNA methylase (ArmA) in resistance of Acinetobacter baumannii clinical isolates against aminoglycosides. Rev. Soc Bras. Med. Trop. 54, e05992020. doi: 10.1590/0037-8682-0599-2020 33533819 PMC7849326

[B34] Karampatakis T. Tsergouli K. Behzadi P. (2024). Pan-genome plasticity and virulence factors: a natural treasure trove for Acinetobacter baumannii. Antibiotics (Basel) 13, 257. doi: 10.3390/antibiotics13030257 38534692 PMC10967457

[B35] Kaviani R. Pouladi I. Niakan M. Mirnejad R. (2020). Molecular detection of AdeFG efflux pump genes and their contribution to antibiotic resistance in Acinetobacter baumannii clinical isolates. Rep. Biochem. Mol. Biol. 8, 413–418. 32582800 PMC7275827

[B36] Khan M. A. S. Chaity S. C. Hosen M. A. Rahman S. R. (2024). Genomic epidemiology of multidrug-resistant clinical Acinetobacter baumannii in Bangladesh. Infect. Genet. Evol. 123, 105656. doi: 10.1016/j.meegid.2024.105656 39116952

[B37] Kishii K. Hamada M. Aoki K. Ito K. Onodera J. Ishii Y. . (2020). Differences in biofilm formation and transcription of biofilm-associated genes among Acinetobacter baumannii clinical strains belonging to the international clone II lineage. J. Infect. Chemother. 26, 693–698. doi: 10.1016/j.jiac.2020.02.017 32249162

[B38] Li S. Jiang G. Wang S. Wang M. Wu Y. Zhang J. . (2025). Emergence and global spread of a dominant multidrug-resistant clade within Acinetobacter baumannii. Nat. Commun. 16, 2787. doi: 10.1038/s41467-025-58106-9 40118837 PMC11928498

[B39] Lima L. K. O. L. Pinto J. C. G. Misael L. S. Castro R. B. Coelho D. D. Benevides D. V. L. . (2019). Evaluation of contamination by Acinetobacter spp. in an intensive care unit. Rev. Epidemiol. Controle Infect. 9, 241–247. doi: 10.17058/reci.v9i3.12510

[B40] Magiorakos A. P. Srinivasan A. Carey R. B. Carmeli Y. Falagas M. E. Giske C. G. . (2012). Multidrug-resistant, extensively drug-resistant and pandrug-resistant bacteria: an international expert proposal for interim standard definitions for acquired resistance. Clin. Microbiol. Infect. 18, 268–281. doi: 10.1111/j.1469-0691.2011.03570.x 21793988

[B41] Mateo Estrada V. Castillo Ramírez S. (2026). “ Global epidemiology. In: Acinetobacter baumannii [Internet]. Cuernavaca: Programa de Genómica Evolutiva, Centro de Ciencias Genómicas, Universidad Nacional Autónoma de México. Available online at: https://www.acinetobacterbaumannii.no/overview/global-epidemiology/ (Accessed February 3, 2026).

[B42] Mohamed E. A. Raafat M. M. Mohamed R. S. Ali A. E. E. (2023). Acinetobacter baumannii biofilm and its potential therapeutic targets. Future J. Pharm. Sci. 9, 25. doi: 10.1186/s43094-023-00525-w 38164791

[B43] Muthuraman V. Roy P. Dean P. Lopes B. S. Shehreen S. (2026). The balance between defence systems and horizontal gene transfer shapes adaptation in clinical strains of Acinetobacter spp. J. Appl. Microbiol. 137, lxag069. doi: 10.1093/jambio/lxag069 41790112

[B44] National Center for Biotechnology Information (2025a). Acinetobacter baumannii ASM250414v1. RefSeq Genome Database. Bethesda: National Library of Medicine (US), National Center for Biotechnology Information. Available online at: https://www.ncbi.nlm.nih.gov/datasets/genome/GCF_002504145.1.

[B45] National Center for Biotechnology Information (2025b). Acinetobacter baumannii ASM326427v1. In: RefSeq Genome Database [Internet]. Bethesda: National Library of Medicine (US), National Center for Biotechnology Information. Available online at: https://www.ncbi.nlm.nih.gov/datasets/genome/GCF_003264275.1.

[B46] Nie X. M. Huang P. H. Ye Q. F. Wan Q. Q. (2015). The distribution, drug resistance, and clinical characteristics of Acinetobacter baumannii infections in solid organ transplant recipients. Transplant. Proc. 47, 2860–2864. doi: 10.1016/j.transproceed.2015.09.037 26707303

[B47] Pan S. Liu K. Huang Y. Gong Y. Wang J. Lei Z. . (2025). Epidemiology and molecular typing of multidrug-resistant Acinetobacter baumannii burn wound isolates from six Chinese provinces. Sci. Rep. 15, 45076. doi: 10.1038/s41598-025-31214-8 41430092 PMC12749299

[B48] Pearl S. Anbarasu A. (2025). Genomic landscape of nosocomial Acinetobacter baumannii: a comprehensive analysis of the resistome, virulome, and mobilome. Sci. Rep. 15, 18203. doi: 10.1038/s41598-025-03246-7 40414962 PMC12104467

[B49] Rashvand P. Peymani A. Mohammadi M. Karami A. A. Samimi R. Hajian S. . (2021). Molecular survey of aminoglycoside-resistant Acinetobacter baumannii isolated from tertiary hospitals in Qazvin, Iran. New Microbes New Infect. 42, 100883. doi: 10.1016/j.nmni.2021.100883 34094583 PMC8165567

[B50] Rodrigues D. C. S. Silveira M. C. Pribul B. R. Karam B. R. S. Picão R. C. Kraychete G. B. . (2024). Genomic study of Acinetobacter baumannii strains co-harboring blaOXA-58 and blaNDM-1 reveals a large multidrug-resistant plasmid encoding these carbapenemases in Brazil. Front. Microbiol. 15, 1439373. doi: 10.3389/fmicb.2024.1439373 39086650 PMC11288812

[B51] Roy S. Chowdhury G. Mukhopadhyay A. K. Dutta S. Basu S. (2022). Convergence of biofilm formation and antibiotic resistance in Acinetobacter baumannii infection. Front. Med. (Lausanne) 9, 793615. doi: 10.3389/fmed.2022.793615 35402433 PMC8987773

[B52] Sahayarayan J. J. Thiyagarajan R. Prathiviraj R. Tn K. Rajan K. S. Manivannan P. . (2024). Comparative genome analysis reveals putative and novel antimicrobial resistance genes common to the nosocomial infection pathogens. Microb. Pathog. 197, 107028. doi: 10.1016/j.micpath.2024.107028 39426637

[B53] Shaikh A. Marathe A. Prajapati B. (2025). Genomic analysis of virulence factors of nosocomial multidrug-resistant Acinetobacter baumannii from a tertiary care hospital. Microbiol. Spectr. 13, e0070725. doi: 10.1128/spectrum.00707-25 41217201 PMC12671222

[B54] Sheldon J. R. Skaar E. P. (2020). Acinetobacter baumannii can use multiple siderophores for iron acquisition, but only acinetobactin is required for virulence. PloS Pathog. 16, e1008995. doi: 10.1371/journal.ppat.1008995 33075115 PMC7595644

[B55] Shenkutie A. M. Yao M. Z. Siu G. K. Wong B. K. C. Leung P. H. (2020). Biofilm-induced antibiotic resistance in clinical Acinetobacter baumannii isolates. Antibiotics (Basel) 9, 817. doi: 10.3390/antibiotics9110817 33212840 PMC7698371

[B56] Sidor-Dzitkowska K. Marszalik A. Kraśnicka A. Zalewska M. Wróblewska M. Bakuła Z. . (2025). Genotyping of Acinetobacter baumannii clinical isolates from Polish hospitals uncovers novel sequence types. Sci. Rep. 15, 40171. doi: 10.1038/s41598-025-23877-0 41249314 PMC12623429

[B57] Silva-Santana G. Baêta Júnior E. S. Silva Conceição G. M. Aguiar-Alves F. Lima Brandão M. L. Lopes-Torres E. J. . (2025a). Intervention of Corynebacterium striatum in the sessile lifestyle of Staphylococcus aureus wild-type and mutants for ica genes in polymicrobial biofilms. Microb. Pathog. 204, 107577. doi: 10.1016/j.micpath.2025.107577 40222568

[B58] Silva-Santana G. da Conceição G. M. S. Brandão M. L. L. Mattos-Guaraldi A. L. Júnior R. H. (2025b). Standardized method for quantifying colony-forming units in Corynebacterium striatum and Staphylococcus aureus biofilms on hydrophilic and hydrophobic surfaces. J. Microbiol. Methods 237, 107216. doi: 10.1016/j.mimet.2025.107216 40784432

[B59] Stoian I. A. Balas Maftei B. Florea C. E. Rotaru A. Costin C. A. Pasare M. A. . (2025). Multidrug-resistant Acinetobacter baumannii: resistance mechanisms, emerging therapies, and prevention—a narrative review. Antibiotics (Basel) 15, 2. doi: 10.3390/antibiotics15010002 41594040 PMC12837888

[B60] Sun X. Ni Z. Tang J. Ding Y. Wang X. Li F. (2021). The AbaI/AbaR quorum sensing system effects on pathogenicity in Acinetobacter baumannii. Front. Microbiol. 12, 679241. doi: 10.3389/fmicb.2021.679241 34322102 PMC8312687

[B61] Supreeta M. S. Parameshwari K. K. Girija A. S. S. Priyadharsini J. V. (2025). Molecular characterization of pga gene types A–D among multidrug-resistant strains of Acinetobacter baumannii. Indian J. Med. Microbiol. 15, 536–542. doi: 10.15789/2220-7619-MCO-17785

[B62] Tang J. Chen Y. Wang X. Ding Y. Sun X. Ni Z. (2020). Contribution of the AbaI/AbaR quorum sensing system to resistance and virulence of Acinetobacter baumannii clinical strains. Infect. Drug Resist. 13, 4273–4281. doi: 10.2147/IDR.S276970 33262621 PMC7699449

[B63] Tomaras A. P. Dorsey C. W. Edelmann R. E. Actis L. A. (2003). Attachment to and biofilm formation on abiotic surfaces by Acinetobacter baumannii: involvement of a novel chaperone-usher pili assembly system. Microbiol. (Reading) 149, 3473–3484. doi: 10.1099/mic.0.26541-0 14663080

[B64] Wang T. H. Leu Y. S. Wang N. Y. Liu C. P. Yan T. R. (2018). Prevalence of different carbapenemase genes among carbapenem-resistant Acinetobacter baumannii blood isolates in Taiwan. Antimicrob. Resist. Infect. Control 7, 123. doi: 10.1186/s13756-018-0410-5 30338061 PMC6182870

[B65] Wareth G. Brandt C. Sprague L. D. Neubauer H. Pletz M. W. (2021a). WGS based analysis of acquired antimicrobial resistance in human and non-human Acinetobacter baumannii isolates from a German perspective. BMC Microbiol. 21, 210. doi: 10.1186/s12866-021-02270-7 34243717 PMC8272256

[B66] Wareth G. Linde J. Nguyen N. H. Nguyen T. N. M. Sprague L. D. Pletz M. W. . (2021b). WGS-based analysis of carbapenem-resistant Acinetobacter baumannii in Vietnam and molecular characterization of antimicrobial determinants and MLST in Southeast Asia. Antibiotics (Basel) 10, 563. doi: 10.3390/antibiotics10050563 34064958 PMC8150915

[B67] Wimalasekara R. L. White D. Kumar A. (2025). Targeting Acinetobacter baumannii resistance-nodulation-division efflux pump transcriptional regulators to combat antimicrobial resistance. NPJ Antimicrob. Resist. 3, 4. doi: 10.1038/s44259-024-00074-z 39863717 PMC11762787

[B68] Woo P. C. Woo G. K. Lau S. K. Wong S. S. Yuen K. Y. (2002). Single gene target bacterial identification: groEL gene sequencing for discriminating clinical isolates of Burkholderia pseudomallei and Burkholderia Thailandensis. Diagn. Microbiol. Infect. Dis. 44, 143–149. doi: 10.1016/s0732-8893(02)00439-x 12458120

[B69] World Health Organization (2024). “ WHO updates list of drug-resistant bacteria most threatening to human health,” in World Health Organization ( World Health Organization, Geneva).

[B70] Xiong L. Yi F. Yu Q. Huang X. Ao K. Wang Y. . (2022). Transcriptomic analysis reveals the regulatory role of quorum sensing in Acinetobacter baumannii ATCC 19606 via RNA-seq. BMC Microbiol. 22, 198. doi: 10.1186/s12866-022-02612-z 35971084 PMC9380347

[B71] Zhang T. Xu X. Xu C. F. Bilya S. R. Xu W. (2021). Mechanical ventilation-associated pneumonia caused by Acinetobacter baumannii in Northeast China region: analysis of genotype and drug resistance of bacteria and patients' clinical features over 7 years. Antimicrob. Resist. Infect. Control 10, 135. doi: 10.1186/s13756-021-01005-7 34526127 PMC8444615

[B72] Zueter A. R. M. Balawi H. A. Balawi D. A. Al-Tamimi M. Sawan H. M. Binsuwaidan R. . (2025). Genetic diversity and phylogenetic analysis of carbapenem-resistant Acinetobacter baumannii clinical isolates in Jordan. Acta Trop. 268, 107708. doi: 10.1016/j.actatropica.2025.107708 40562185

